# The Global, Regional, and National Burden and Trends of Breast Cancer From 1990 to 2019: Results From the Global Burden of Disease Study 2019

**DOI:** 10.3389/fonc.2021.689562

**Published:** 2021-05-21

**Authors:** Shangbo Xu, Yiyuan Liu, Taofeng Zhang, Jiehua Zheng, Weixun Lin, Jiehui Cai, Juan Zou, Yaokun Chen, Yanna Xie, Yexi Chen, Zhiyang Li

**Affiliations:** ^1^ Department of Internal Medicine, People’s Hospital of Jieyang, Jieyang Hospital Affiliated to SunYat-sen University, Jieyang, China; ^2^ Department of General Surgery, The Second Affiliated Hospital of Shantou University Medical College, Shantou, China; ^3^ Department of Breast Disease Research Center, The Medical Research Institute of Shantou Doctoral Association, Shantou, China; ^4^ Department of Internal Medicine, The First Affiliated Hospital of Shantou University Medical College, Shantou, China

**Keywords:** breast cancer, global cancer burden, incidence, mortality, risk factor

## Abstract

**Background:**

The burden of breast cancer has been increasing globally. The epidemiology burden and trends need to be updated. This study aimed to update the burden and trends of breast cancer incidences, deaths, and disability-adjusted life-years (DALYs) from 1990 to 2019, using the Global Burden of Disease 2019 study.

**Methods:**

The data of incidences, deaths, DALYs, and age-standardized rates were extracted. Estimated annual percentage changes were used to quantify the trends of age-standardized rates. Besides, the population attributable fractions of the risk factors of breast cancer were also estimated.

**Results:**

Globally, the incidences of breast cancer increased to 2,002,354 in 2019. High social-development index (SDI) quintiles had the highest incidence cases with a declining trend in age-standardized incidence rate. In 2019, the global deaths and DALYs of breast cancer increased to 700,660 and 20,625,313, respectively. From 1990 to 2019, the age-standardized mortality rates and age-standardized DALY rates declined globally, especially in high and high-middle SDI quintiles. Besides, the trends varied from different regions and countries. The proportion of the patients in the 70+ years age group increased globally. Deaths of breast cancer attributable to high fasting plasma glucose and high body mass index increased globally, and high fasting plasma glucose was the greatest contributor to the global breast cancer deaths.

**Conclusion:**

The burden of breast cancer in higher SDI quintiles had gone down while the burden was still on the rise in lower SDI quintiles. It is necessary to appeal to the public to decrease the exposure of the risk factors.

## Introduction

It has been estimated that breast cancer surpassed lung cancer as the first cause of global cancer incidence in females, with 2,261,419 new cases in 2020 (1). Moreover, breast cancer is the fifth leading cause of cancer death in females and also a considerable cause of cancer death in males worldwide (1, 2). The incidence rate of breast cancer varies in different countries, which is commonly higher in high income countries than in low- middle income countries (1, 3). Although the incidence keeps increasing, the survival rate of global breast cancer has been improved during the past three decades (3).However, the mortality also differs throughout the world (3, 4), which even increases rapidly in some locations (5). Thus, data concerning the breast cancer burden and trends in different regions and countries is important for decision-making and resource allocation.

The Global Burden of Disease (GBD) study estimates a variety of metrics of diseases annually since1990, which provides a great opportunity for comparable assessment of the breast cancer burden and trends at the global, regional and national levels. In addition, the GBD study also estimates the population attributable fractions of risk factors related to breast cancer that may be useful for policy makers to identify intervention priorities for public health actions. Researchers have presented the estimation of the global burden of breast cancer based on the GBD study 2017 (5, 6). Recently, the GBD study 2019, a comprehensive update of epidemiology levels, has incorporated new datasets, enhanced method performance and standardization, and reflected developments in scientific understanding (7, 8). Nevertheless, there has been no study analyzing the breast cancer burden and trends based on the GBD study 2019 until now. Hence, this study aims to describe and compare the variations in breast cancer burden and its attributable risk factors between 1990 and 2019 at the global, regional, and national levels.

## Materials and Methods

### Data Sources

Data about the breast cancer burden were collected from GBD 2019, which systematically and comprehensively estimates the burden of 359 diseases and injuries and 84 risk factors across ages, sexes, regions and 204 countries and territories from 1990 to 2019 (7, 8). Herein, data about breast cancer’s incidence, death, disability-adjusted life-year (DALY), and their corresponding respective age-standardized rates (ASRs) from 1990 to 2019 were available at the Global Health Data Exchange query tool (http://ghdx.healthdata.org/gbd-results-tool). Meanwhile, the information about the distributions of age was also acquired. To be consistent with previous articles, socio-Demographic index (SDI), which is a comprehensive measurement of educational level, per capita income, and total fertility, was used to group the countries into five SDI quintiles (low, low-middle, middle, high-middle, and high levels). Moreover, the world was separated into 21 regions in terms of geography.

### Estimation Framework

Detailed methodology of GBD study 2019 was described in two previous studies (7, 8). In brief, mortality data for breast cancer was based on vital registration, verbal autopsy and cancer registry. Mortality-to-incidence ratios (MIRs) were generated by using sources that both incidence and mortality for the same year were available. MIRs were initially modeled with a linear-step mixed-effects model. Then, the estimates from this model were smoothed over space and time, and adjusted by spatiotemporal Gaussian process regression. Both observed and estimated mortalities (computed from MIRs and incidence data) were used as inputs for a Cause of Death Ensemble model (9). The CoDCorrect algorithm adjusted single cause estimates to make sure that all single causes summed to the estimated all-cause mortality. The final mortality was divided by the estimated MIRs to calculate breast cancer incidence. Prevalence was divided into four different health states, including diagnosis or treatment, remission, metastatic, and terminal phase. Years lived with disability (YLDs) were estimated by multiplying these health states by corresponding disability weights, while years of life lost (YLLs) were calculated by multiplying the estimated number of deaths by age with a standard life expectancy at corresponding age. And DALYs were computed by summing YLDs and YLLs.

### Estimation of Attributable Burden

The GBD 2019 used the comparative risk assessment framework to quantify the associations between the burden of diseases and impairments and 84 environmental, occupational, metabolic, and behavioral risk factors (8, 10). Population attributable fractions were derived from systematic reviews of the literature. We reported the percentage of death due to breast cancer that were attributable to the following six risk factors: alcohol use, high body mass index (BMI), high fasting plasma glucose (FPG), low physical activity, diet high in red meat and tobacco.

### Statistical Analysis

When comparing populations from different locations or a sample population over time, it was considered imperative to standardize the data. The ASRs, including age-standardized incident rates (ASIRs), age-standardized mortality rates (ASMRs), age-standardized DALY rates, were calculated in accordance with the direct method by summing up the products of age-specific rates (α*_i_*, where *i* denotes the *i*th age class) and the number of persons (or weight) (w*i*) in the same age subgroup *i* of the chosen reference standard population, then dividing the sum of the standard population weights, i.e (11).

$$ASR=\frac{\Sigma_{i=1}^{A}a_{i}w_{i}}{\Sigma_{i=1}^{A}a_{i}}\times 100,00$$

Moreover, the trends of ASRs provided clues for the dynamic changes of risk factors, as well as surrogates for shifting disease patterns in the population. The estimated annual percentage changes (EAPCs) were used to track the ASRs trends over a specified time interval. A linear regression line was fitted to the natural logarithm of the rates with calendar year as an independent variable i.e., y = α + βx + ε, where x referred to calendar year, and y presented ln(ASR). Then EAPC could be calculated as 100 × (10^β − 1^), and its 95% confidential interval (CI) also could be computed from the linear regression model. The ASRs were considered to be in a decreasing trend when EAPC and the 95%CI were <0. On the contrary, the ASRs were deemed to be on the rise when EAPC and the 95%CI were > 0. In addition, we analyzed the correlation between EAPC and SDI values and ASRs by using the Pearson’s correlation test model. The 95% uncertainty intervals (UIs) were calculated as taking the 2.5th and 97.5th values of 1,000 draw-level estimates for each quantity of interest. All statistical analyses were conducted by R program (Version 3.6.1), with a P value <0.05 considered statistically significant.

## Results

### Breast Cancer Incidence Burden

Globally, there were 2,002,354 (95% UI: 1,832,150–2,172,540) new cases of breast cancer, with an ASIR of 24.17 per 100,000 persons (95% UI: 22.11–26.24) in 2019 (**Table 1**). The number of incidence cases increased by 128.32% (95% UI: 109.45–147.49%) from 1990 to 2019, and ASIR showed a slightly increasing trend in this period (EAPC 0.32, 95% CI: 0.27–0.38) (**Table 2**). As for SDI, both the incidence cases and ASIR were significantly lower in lower SDI levels. From 1990 to 2019, the incidence cases increased in all SDI quintiles, precipitously increased in middle SDI quintiles (2.90-fold) and slowly in high SDI quintiles (0.58-fold) (**Table 2**). With the exception of high SDI quintiles (EAPC −0.37, 95% CI: −0.37–0.17), ASIR increased in other four SDI quintiles, with the sharpest increase in middle SDI quintiles (EAPC 1.95, 95% CI: 1.92–1.98) (**Table 2**).

**Table 1:** Breast cancer incidence cases, age-standardized incidence rate, deaths, age-standardized mortality rate, DALYs, and age-standardized DALY rates in 2019.

Characteristics	Incidence cases (95%UI)	ASIR per 10^5^ (95% UI)	Deaths (95% UI)	ASMR per 10^5^ (95% UI)	DALYs (95% UI)	Age-standardized DALY rates per 10^5^ (95% UI)
**Global**	2,002,354 (1,832,150–2,172,540)	24.17 (22.11–26.24)	700,660 (647,384–751,555)	8.62 (7.95–9.25)	2,0625,313 (19,043,049–22,174,397)	247.63 (228.68–266.08)
**Sex**						
Male	25,143 (22,231–27,786)	0.65 (0.58–0.72)	12,099 (10,693–13,322)	0.33 (0.29–0.36)	315,126 (278,546–349,292)	7.96 (7.03–8.80)
Female	1,977,212 (1,807,615–2,145,215)	45.86 (41.91–49.76)	688,562 (635,323–739,571)	15.88 (14.66–17.07)	20,310,187 (18,744,799–21,866,646)	473.83 (437.30–510.51)
**SDI**						
High	678,945 (606,880–753,696)	41.22 (36.88–45.65)	167,553 (151,809–176,807)	9.05 (8.36–9.47)	4,083,850 (3,816,265–4,354,647)	252.68 (237.71–269.32)
High-middle	516,502 (464,353–574,104)	26.00 (23.34–28.88)	165,934 (152,738–179,587)	8.31 (7.63–9.00)	4,570,253 (4,209,331–4,954,003)	230.43 (212.17–249.87)
Middle	492,444 (436,591–552,088)	18.49 (16.40–20.73)	184,787 (166,334–205,342)	7.30 (6.60–8.12)	5,923,359 (5,318,524–6,558,618)	219.85 (197.35–243.15)
Low-middle	230,747 (202,809–259,274)	15.38 (13.54–17.27)	127,410 (110,474–144,919)	8.94 (7.77–10.16)	4,194,850 (3,622,482–4,785,991)	271.67 (235.41–309.31)
Low	81,318 (70,923–92,857)	13.42 (11.85–15.14)	54,467 (47,490–62,057)	9.83 (8.59–11.12)	1,837,382 (1,599,786–2,101,742)	283.77 (248.24–323.37)
**Region**						
Central Asia	17,838 (15,855–20,014)	21.11 (18.88–23.51)	7,577 (6,791–8,460)	9.85 (8.87–10.89)	247,812 (219,124–279,602)	285.88 (254.39–321.26)
Central Europe	61,583 (53,370–70,791)	32.49 (28.13–37.48)	23,351 (20,388–26,568)	11.32 (9.86–12.93)	563,173 (490,262–647,228)	298.04 (257.73–344.01)
Eastern Europe	94,947 (81,403–111,153)	29.83 (25.53–35.06)	35,368 (30,675–40,861)	10.59 (9.17–12.27)	966,186 (840,188–1,123,048)	303.90 (263.08–354.02)
Australasia	19,268 (15,570–23,873)	44.31 (35.73–54.87)	4,481 (4,032–4,829)	9.36 (8.60–10.03)	113,906 (104,805–124,260)	266.02 (246.17–288.90)
High-income Asia Pacific	97,591 (81,436–115,490)	28.81 (24.14–34.27)	20,687 (17,891–22,507)	5.18 (4.68–5.53)	537,969 (488,067–585,815)	164.19 (152.45–178.25)
High-income North America	283,122 (236,467–337,819)	49.88 (41.65–59.83)	61,594 (56,936–64,844)	10.04 (9.39–10.51)	1,563,466 (1,464,038–1,670,115)	282.40 (265.58–301.63)
Southern Latin America	24,765 (19,368–31,351)	30.81 (23.98–39.10)	11,243 (10,404–12,013)	13.60 (12.62–14.52)	277,842 (259,691–298,889)	348.56 (326.22–374.16)
Western Europe	341,500 (295,017–390,202)	45.15 (38.99–51.84)	98,409 (88,188–104,248)	10.90 (10.01–11.45)	2,184,732 (2,032,712–2,339,874)	290.47 (272.68–311.07)
Andean Latin America	9,052 (7,337–11,139)	15.45 (12.56–19.02)	3,818 (3,143–4,686)	6.69 (5.52–8.18)	114,444 (92,542–142,511)	193.23 (156.52–239.86)
Caribbean	15,101 (12,760–17,754)	29.26 (24.67–34.37)	5,804 (4,940–6,788)	11.22 (9.53–13.11)	168,795 (140,179–199,784)	328.29 (272.00–389.37)
Central Latin America	50,822 (42,750–60,336)	20.59 (17.30–24.42)	16,830 (14,445–19,743)	6.98 (6.00–8.18)	520,817 (441,173–614,900)	208.72 (177.04–246.25)
Tropical Latin America	53,821 (50,463–57,171)	21.53 (20.17–22.89)	20,661 (19,269–21,895)	8.46 (7.87–8.97)	614,365 (578,255–652,011)	243.80 (229.51–258.62)
North Africa and Middle East	96,190 (83,600–110,516)	18.44 (16.10–21.10)	36,214 (31,423–41,545)	7.62 (6.66–8.68)	1,244,209 (1,071,622–1,438,412)	232.28 (201.38–267.48)
South Asia	219,120 (181,404–260,611)	14.17 (11.76–16.82)	127,800 (105,469–151,677)	8.72 (7.23–10.31)	4,218,058 (3,461,522–5,024,210)	264.62 (218.07–314.62)
East Asia	389,533 (309,987–484,103)	18.37 (14.63–22.79)	101,023 (82,358–122,871)	4.91 (3.99–5.94)	3,106,704 (2,558,341–3,748,533)	146.32 (120.33–176.17)
Oceania	3,002 (2,295–3,852)	32.05 (24.68–40.73)	1,810 (1,385–2,297)	21.04 (16.32–26.53)	68,335 (51,802–88,227)	690.80 (530.35–880.44)
Southeast Asia	139,786 (120,078–162,660)	20.26 (17.50–23.45)	67,276 (57,787–77,241)	10.38 (8.98–11.85)	2,286,684 (1,961,049–2,656,738)	324.34 (278.99–374.73)
Central Sub-Saharan Africa	9,883 (7,105–13,015)	16.15 (11.65–21.41)	6,988 (5,085–9,177)	12.87 (9.37–16.91)	233,055 (169,283–306,210)	341.73 (247.53–449.21)
Eastern Sub-Saharan Africa	25,554 (21,604–29,702)	13.76 (11.88–15.69)	17,823 (15,241–20,467)	10.74 (9.29–12.13)	575,320 (481,857–672,688)	281.40 (240.11–325.38)
Southern Sub-Saharan Africa	11,732 (10,429–13,151)	19.73 (17.64–22.03)	7,259 (6,449–8,089)	13.33 (12.02–14.77)	210,438 (185,318–239,360)	335.75 (297.05–378.56)
Western Sub-Saharan Africa	38,143 (29,623–47,064)	17.30 (13.63–21.07)	24,643 (19,341–30,965)	12.27 (9.85–15.09)	809,001 (622,213–1,030,814)	341.76 (266.08–429.39)

ASIR, age-standardized incident rates; ASMR, age-standardized mortality rates; DALY, disability adjusted life-year; SDI, socio-demographic index; UI, uncertain interval.

**Table 2:** The trends in incidence, mortality and DALYs of breast cancer between 1990 and 2019.

Characteristics	Relative change in incident cases (95% UI)	EAPC of ASIR (95% CI)	Relative change in deaths (95% UI)	EAPC of ASMR (95% CI)	Relative change in DALYs (95% UI)	EAPC of age-standardized DALY rates (95% CI)
**Global**	128.32% (109.45–147.49%)	0.32 (0.27–0.38)	83.95% (70.07–96.74%)	−0.56 (−0.60–0.51)	76.57% (62.25–89.28%)	−0.51(−0.56–0.45)
**Sex**						
Male	168.27% (130.76–202.48%)	0.90 (0.75–1.07)	105.42% (75.10–133.74%)	−0.23 (−0.37–0.09)	104.13% (73.23–132.99%)	0.14 (−0.01–0.29)
Female	127.89% (108.89–147.27%)	0.36 (0.31–0.42)	83.61% (69.63–96.84%)	−0.51 (−0.56–0.46)	76.20% (61.90–89.45%)	−0.51 (−0.57–0.45)
**SDI**						
High	57.50% (41.28–74.19%)	−0.27 (−0.37–0.17)	21.91% (15.77–26.74%)	−1.52 (−1.57–1.47)	9.96% (5.35–14.41%)	−1.53(−1.58–1.48)
High-middle	126.07% (102.79–152.12%)	0.67 (0.58–0.77)	58.73% (47.59–72.08%)	−0.84 (−0.96–0.71)	44.20% (33.26–56.90%)	−0.99(−1.10–0.87)
Middle	290.94% (238.51–347.55%)	1.95 (1.92–1.98)	154.39% (123.62–186.32%)	0.29 (0.26–0.32)	136.36% (107.24–165.35%)	0.25(0.22–0.29)
Low-middle	249.10% (194.40–313.10%)	1.54 (1.44–1.65)	173.88% (125.03–230.69%)	0.63 (0.54–0.72)	159.34% (114.30–210.56%)	0.59(0.49–0.71)
Low	227.19% (170.38–297.28%)	1.29 (1.23–1.35)	179.11% (127.48–234.90%)	0.75 (0.71–0.80)	175.70% (125.96–231.75%)	0.67(0.61–0.73)
**Region**						
Central Asia	81.37% (61.25–104.93%)	0.33 (0.29–0.37)	45.17% (29.96–61.98%)	−0.22 (−0.31–0.13)	44.68% (28.91–63.13%)	−0.58 (−0.68–0.48)
Central Europe	68.58% (47.83–92.33%)	0.88 (0.75–1.02)	32.86% (16.47–50.21%)	−0.34 (−0.44–0.25)	11.34% (−2.87–26.52%)	−0.63 (−0.72–0.54)
Eastern Europe	51.16% (30.08–75.70%)	0.68 (0.51–0.85)	17.84% (3.05–35.76%)	−0.60 (−0.88–0.31)	5.87% (−7.32–22.43%)	−0.87 (−1.16–0.57)
Australasia	89.59% (54.34–134.22%)	−0.29 (−0.46–0.11)	37.35% (27.28–47.38%)	−1.67 (−1.77–1.55)	22.81% (14.68–32.10%)	−1.71 (−1.80–1.61)
High-income Asia Pacific	173.00% (129.91–223.34%)	2.03 (1.79–2.28)	115.59% (93.01–131.88%)	0.45 (0.31–0.60)	67.85% (56.76–78.90%)	0.31 (0.14–0.47)
High-income North America	36.38% (14.78–63.89%)	−1.05 (−1.16–0.95)	16.47% (11.53–21.20%)	−1.70 (−1.79–1.61)	6.47% (1.72–11.28%)	−1.84 (−1.95–1.74)
Southern Latin America	104.43% (59.23–157.39%)	0.38 (0.23–0.54)	55.28% (45.19–65.28%)	−0.72 (−0.84–0.60)	39.09% (29.62–48.87%)	−0.87 (−0.95–0.77)
Western Europe	48.25% (29.66–69.63%)	−0.05 (−0.21–0.12)	10.63% (4.00–15.51%)	−1.54 (−1.60–1.48)	−4.48% (−8.99–0.10%)	−1.67 (−1.73–1.61)
Andean Latin America	294.07% (209.21–398.96%)	1.37 (1.25–1.48)	162.05% (110.06–228.18%)	−0.18 (−0.29–0.07)	138.04% (88.51–202.35%)	−0.33 (−0.45–0.22)
Caribbean	148.82% (112.15–191.11%)	1.04 (0.96–1.11)	109.76% (80.98–143.39%)	0.32 (0.24–0.41)	97.47% (66.73–130.77%)	0.29 (0.22–0.37)
Central Latin America	323.18% (257.52–406.57%)	1.65 (1.55–1.76)	189.35% (148.24–237.75%)	0.23 (0.16–0.31)	167.18% (126.64–213.95%)	0.24 (0.17–0.31)
Tropical Latin America	213.61% (194.24–235.61%)	0.78 (0.55–1.02)	128.54% (113.73–143.81%)	−0.49 (−0.65–0.32)	109.29% (96.76–123.33%)	−0.47 (−0.63–0.30)
North Africa and Middle East	377.91% (290.48–456.34%)	2.21 (2.15–2.28)	203.72% (145.47–255.82%)	0.68 (0.60–0.75)	197.21% (142.59–247.02%)	0.53 (0.45–0.61)
South Asia	290.99% (203.14–399.75%)	1.64 (1.51–1.77)	210.07% (136.74–303.53%)	0.70 (0.59–0.82)	195.19% (126.59–280.34%)	0.81 (0.69–0.94)
East Asia	354.05% (238.35–496.38%)	2.81 (2.71–2.91)	131.19% (79.32–198.71%)	0.09 (0.03–0.15)	106.88% (61.51–168.21%)	−0.10 (−0.16–0.03)
Oceania	254.60% (167.33–367.75%)	1.26 (1.21–1.30)	217.59% (140.50–320.20%)	0.93 (0.89–0.98)	219.10% (138.57–328.11%)	0.96 (0.91–1.01)
Southeast Asia	211.03% (152.90–275.65%)	1.12 (1.06–1.17)	130.04% (89.56–172.54%)	−0.01 (−0.07–0.06)	115.03% (74.06–159.67%)	−0.10 (−0.16–0.03)
Central Sub-Saharan Africa	233.35% (139.58–356.02%)	1.08 (0.94–1.21)	199.41% (116.41–305.69%)	0.81 (0.70–0.92)	190.21% (104.78–293.04%)	0.55 (0.44–0.66)
Eastern Sub-Saharan Africa	189.78% (129.53–257.34%)	0.81 (0.69–0.93)	150.82% (104.97–204.86%)	0.45 (0.35–0.55)	144.41% (95.64–208.19%)	0.20 (0.09–0.32)
Southern Sub-Saharan Africa	166.03% (131.94–205.61%)	1.35 (1.17–1.52)	141.63% (110.39–177.66%)	1.03 (0.79–1.27)	119.82% (91.65–153.34%)	0.84 (0.61–1.08)
Western Sub-Saharan Africa	268.32% (164.80–392.70%)	1.75 (1.62–1.88)	208.82% (126.26–307.16%)	1.20 (1.08–1.31)	216.89% (131.00–320.71%)	1.19 (1.07–1.31)

ASIR, age-standardized incident rates. ASMR, age-standardized mortality rates. CI, confidential interval. DALY, disability adjusted life-year. EAPC, estimated annual percentage change. SDI, socio-demographic index. UI, uncertain interval.

**Figure 1:**
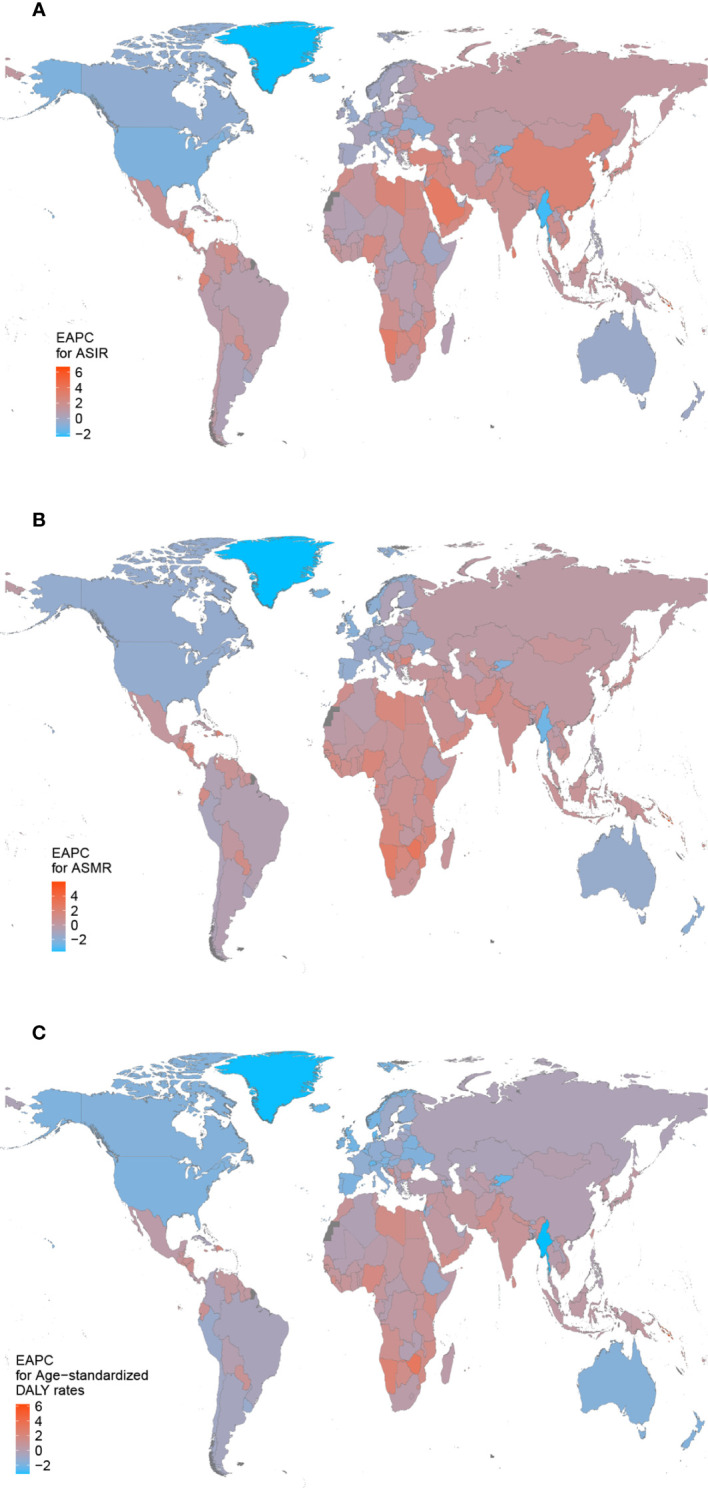
The estimated annual percentage changes (EAPCs) of breast cancer in 204 countries and territories between 1990 and 2019. **(A)** The EAPC of age-standardized incidence rates (ASIR). **(B)** The EAPC of age-standardized mortality rates (ASMR). **(C)** The EAPC of age-standardized disability-adjusted life-year (DALY) rates.

Among 21 GBD regions, East Asia (389,533, 95% UI: 309,987–484,103) had the highest number of breast cancer incidence cases in 2019, while High-income North America had the highest ASIR (49.88, 95% UI: 1.92–2.02 per 100,000 persons) (**Table 1**). From 1990 to 2019, incidence cases of breast cancer increased in all GBD regions, with the most increasing significant trends observed in North Africa and Middle East (377.91%, 95% UI: 290.48–456.34%). Only the High-income North America (EAPC −1.05, 95% CI: −1.16–0.95) and Australasia (EAPC −0.29, 95% CI: −0.46–0.11) showed a declining trend in ASIR during the past three decades. East Asia was observed with a largest increase in the ASIR (EAPC 2.81, 95% CI: 2.71–2.91) (**Table 2**). In 2019, Countries with the highest incidence cases included China (375,484, 95% UI: 296,626–469,983), United States of America (254,486, 95% UI: 210,821–308,184) and India (146,090, 95% UI: 112,452–183,482) (**Table S1** and **Figure S1**). The largest increase between 1990 and 2019 was seen in Solomon Islands (EAPC 6.68, 95% CI: 6.04–7.33). On the contrast, the largest decrease during this period was found in Greenland (EAPC −2.33, 95% CI: −2.60–2.08) (**Figure 1** and **Table S2**).

### Breast Cancer Death Burden

Compared to 1990, the global deaths of breast cancer increased by 83.95% (95% UI: 70.07–96.74%), with 700,660 (95%UI: 647,384–751,555) cases in 2019, but the ASMR decreased to 8.62 per 100,000 persons (95% UI: 7.95–9.25), with an EAPC of −0.56 (95% CI: −0.60–0.51) (**Tables 1** and **2**). Subgroup analysis by SDI quintiles showed that the growth of deaths declined with the elevated SDI level from 1990 to 2019. However, the middle SDI quintiles had the highest number of deaths in 2019 (184,787 (95% UI: 166,334–205,342)) (**Tables 1** and **2**). At the same time, both the high (EAPC −1.52, 95% CI: −1.57–1.47) and high-middle SDI quintiles (EAPC −0.84, 95% CI: −0.96–0.71) were found with a decreasing trend in ASMR (**Table 2**).

Breast cancer deaths mostly occurred in South Asia in 2019 (127,800, 95% UI: 105,469–151,677), while the largest increase of deaths was detected in Oceania, with a 2.17-fold increase (**Table 1**). The ASMR was found to be highest in Oceania in 2019 (21.04 95% UI: 16.32–26.53) (**Table 1**). The ASMR displayed a decreasing trend in nine regions, including High-income North America (EAPC −1.70, 95% CI: −1.79–1.61), Australasia (EAPC −1.67, 95% CI: −1.77–1.55) and Western Europe (EAPC −1.54, 95% CI: −1.60–1.48). Contrarily, the growth speed of ASMR was fasted in Western Sub-Saharan Africa (EAPC 1.20, 95% CI: 1.08–1.31), Southern Sub-Saharan Africa (EAPC 1.03, 95% CI: 0.79–1.27) and Oceania (EAPC 0.93, 95% CI: 0.89–0.98) among the remaining regions (**Table 2**). With the largest populations and highest incidence cases, China (96,306, 95% UI: 77,323–118,090), India (83,510, 95% UI: 64,550–105,994) and United States of America (55,021, 95% UI: 51,008–57,900) had the most breast cancer deaths in 2019 (**Table S1** and **Figure S2**). Solomon Islands (EAPC 5.97, 95% CI: 5.33–6.61) showed the greatest increasing trend of ASMR from 1990 to 2019. By contrast, the largest decreasing trend was seen in Greenland (EAPC −3.64, 95% CI: −4.00–3.27) (**Figure 1** and **Table S2**).

### Breast Cancer DALY Burden

Globally, the breast cancer-related DALYs increased to 20,625,313 (95%UI: 19,043,049–22,174,397) in 2019 (**Table 1**). The DALYs increased in all SDI countries, and the rise was steeper in lower level of SDI. High (EAPC −1.53, 95% CI: −1.58–1.48) and high-middle SDI (EAPC−0.99, 95% CI: −1.10–0.87) exhibited a declining trend in age-standardized DALY rates during the study period (**Table 2**).

For different geographic areas, South Asia (4,218,058, 95% UI: 3,461,522–5,024,210), East Asia (3,106,704, 95% UI: 2,558,341–3,748,533) and Southeast Asia (2,286,684, 95% UI: 1,961,049–2,656,738) had the highest DALY burden in 2019, while Oceania (690.80, 95% UI: 530.35–880.44 per 100,000 persons) had a particularly high age-standardized DALY rates (**Table 1**). From 1990 to 2019, the breast cancer-related DALYs increased in all regions with the exception of Western Europe. Among 11 regions showing a declining trend in age-standardized DALY rates, High-income North America (EAPC −1.84, 95% CI: −1.95–1.74) was the area that had the most rapid decrease. The age-standardized DALY rates displayed a greatest increasing trend in Western Sub-Saharan Africa (EAPC1.20, 95% CI: 1.08–1.31), Oceania (EAPC 0.93, 95% CI: 0.89–0.98) and Southern Sub-Saharan Africa (EAPC 1.03, 95% CI: 0.79–1.27) (**Table 2**). In terms of country or territory, China (2,957,454, 95% UI: 2,408,511–3,590,166), India (2,697,350, 95% UI: 2,090,173–3,424,541) and United States of America (4,218,058, 95% UI: 3,461,522–5,024,210) had the highest DALYs burden in 2019 (**Table S1** and **Figure S3**). Solomon Islands (EAPC 6.27, 95% CI: 5.58–6.96) also showed the greatest increasing trend of age-standardized DALY rates from 1990 to 2019 (**Figure 1** and **Table S2**).

### The Correlation Between ASIR and Both SDI and EAPC

**Figure 2:**
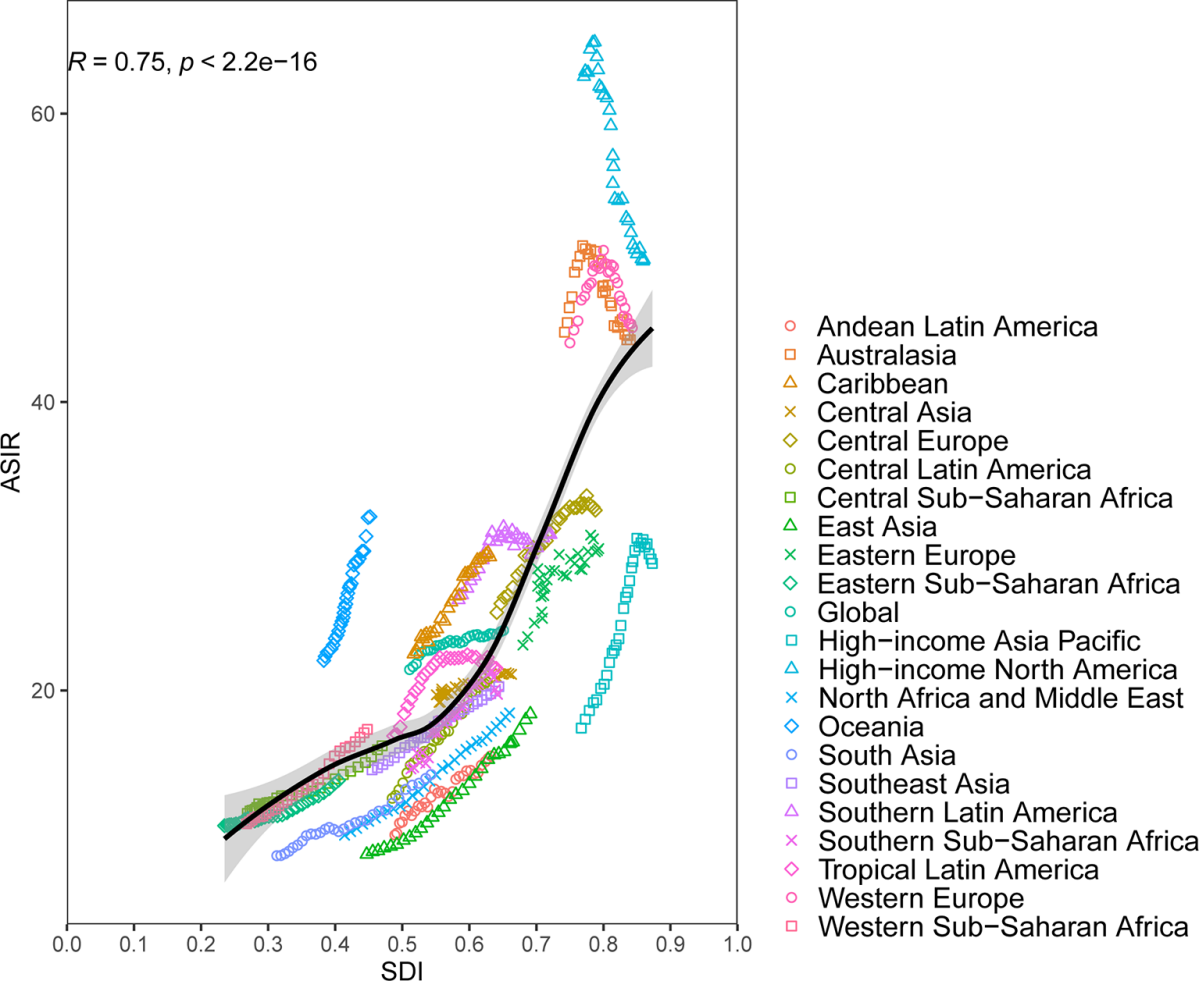
Age-standardized incidence rates (ASIR) for breast cancer for 21 Global Burden of Disease (GBD) regions by Socio-Demographic Index (SDI), 1990–2019; expected values based on SDI and disease rates in all locations are shown as the black line. Thirty points are plotted for each GBD region and show observed ASIR from 1990 to 2017 for that region. The R indices and P value were derived from Pearson correlation analysis.

**Figure 3:**
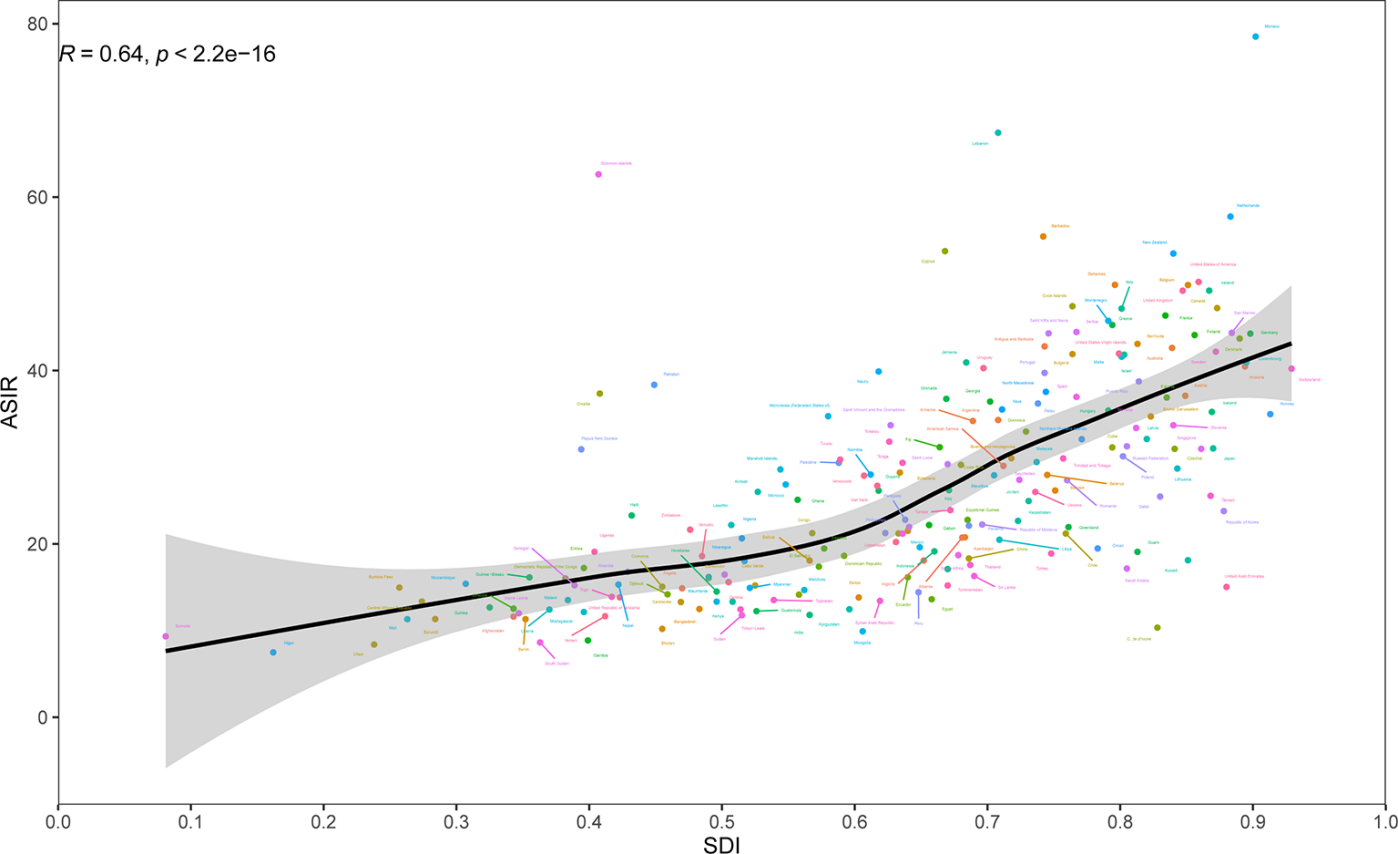
Age-standardized incidence rates (ASIR) of breast cancer by 204 countries and territories and Socio-Demographic Index (SDI), 2019; expected values are shown as the black line. Each point shows observed ASIR for specified country in 2019. The R indices and P value were derived from Pearson correlation analysis.

At the regional-level, a positive association was found between ASIR and the SDI (**Figure 2**). Interestingly enough, among the high-income super region, only High-income Asia Pacific showed an increasing level from 1990 to 2019. Despite having a downtrend of ASIR, high-income North America still showed a higher than expected level of ASIR during the study period. National-level analysis found that there existed a positive correlation between ASIR and the SDI (**Figure 3**). In addition, a negative correlation between EAPC and ASIR in 1990 was observed (**Figure S4**). This indicated that breast cancer cases increased more rapidly in lower incidence countries than in higher incident countries.

### Age-Related Incidence

**Figure 4:**
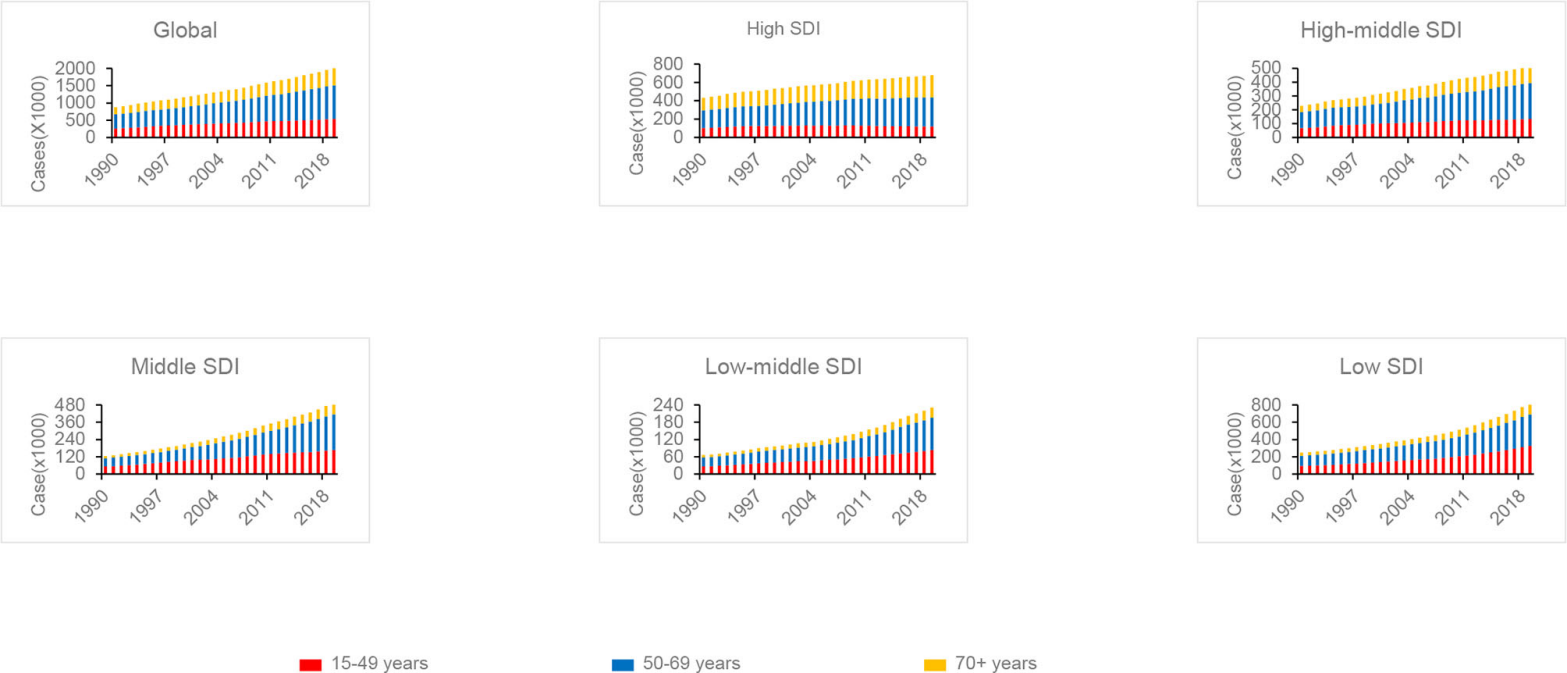
The proportion of the three age groups (15–49 years, 50–69 years, and 70+ years) for breast cancer incidence cases between 1990 and 2019 globally, and in high, high middle, middle, low middle, and low Socio-Demographic Index (SDI) quintiles.

Age distribution was a vital parameter of cancer epidemiology. Therefore, patients were divided into three groups (15–49 years, 50–69 years, and 70+ years) (**Figure 4**). Globally, the proportion of breast cancer patients aged 50–69 years was nearly 49% in 2019, with reaching 56% in Eastern Europe. The ratio of the age 70+ years group was increased with the elevated SDI level. Relatively, the age 70+ years group was higher in the high-income super region. Compared to 1990, the globe and most regions showed an increasing trend of the proportion of the age 70+ years group in 2019. Globally, all age groups showed an increasing trend of incidence cases from 1990 to 2019. However, a declining trend of incidence was observed in the age 15–49 years group after 2004 in the high SDI quintiles.

### Attributable Risk Factors

**Figure 5:**
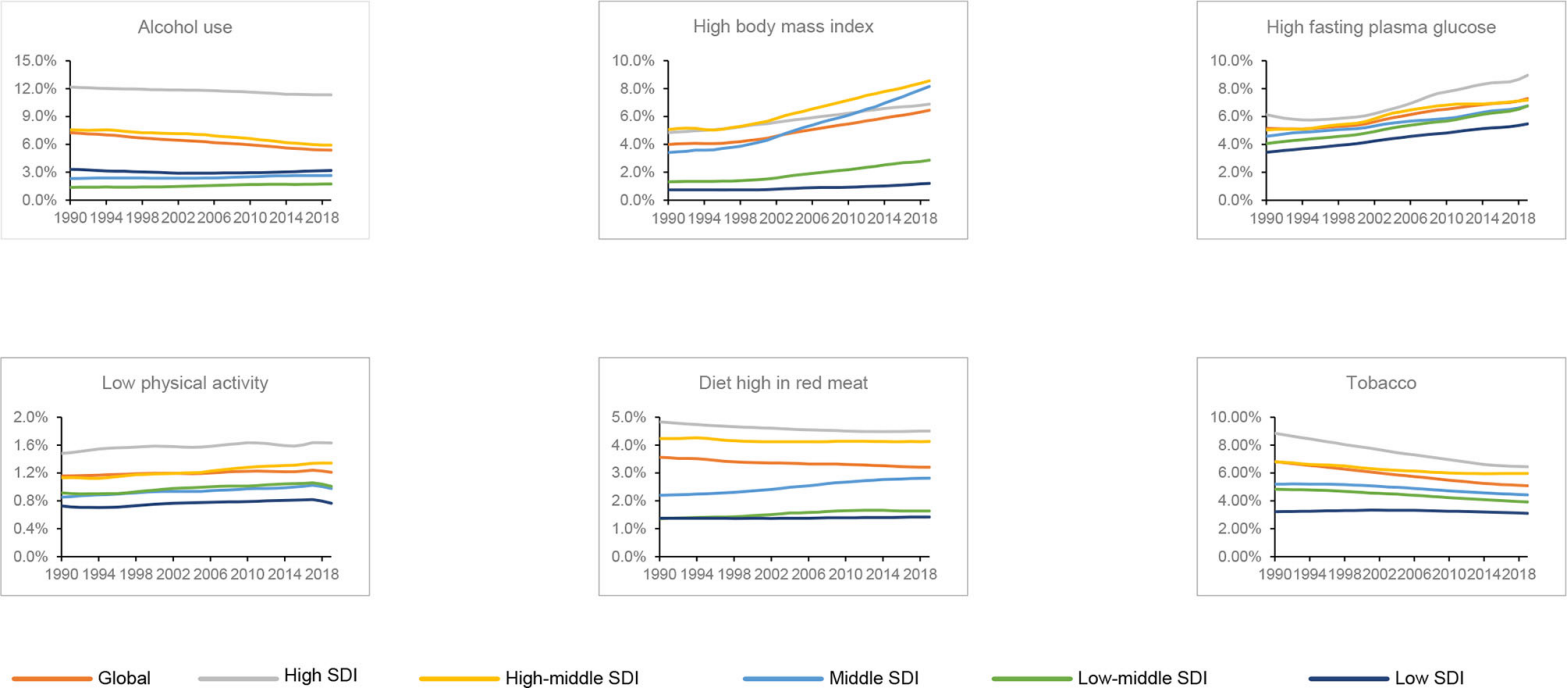
Contributions of different risk factors to breast cancer deaths globally and in five sociodemographic index (SDI) quintiles from 1990 to 2019.

Generally, high FPG was the greatest distributor of deaths in the global level, high SDI, low-middle SDI and low SDI quintiles. The contribution ratio of diet high in red meat was relatively weak. Deaths of breast cancer attributable to high FPG and high BMI increased globally and in all the SDI quintiles between 1990 and 2019, while low physical activity showed with a moderate trend. Except the moderate trend of tobacco and alcohol use attributed deaths in the low SDI quintile, the other SDI quintiles showed decreased trend during study period. Diet high in red meat led to 3.21% of global breast cancer deaths in 2019 with a declining trend between 1990 and 2019. On the contrary, deaths associated with diet high in red meat increased in the low SDI, low-middle SDI and the middle SDI quintiles (**Figure 5**).

## Discussion

Basing on the GBD 2019 results, this research presented the latest comprehensive overview of the global, regional, and national burden of breast cancer and its relevant risk factors. Our results revealed that breast cancer still was a major global public health problem in 2019, and large variations in the trends of incidences, deaths, and DALYs, as well as the percentages of attributable risk factors across regions and countries remained a main epidemiological feature, indicating the importance of a more elaborate delineation of hot spots to the identification of high-risk populations. Although a downtrend of ASIR was detected in High-income North America and some countries, they still reached at a higher than expected level of ASIR during the study period. It is urgent to design a flexible country-tailored approach to effectively decrease the breast cancer burden and counter the imbalance in health care attributable to breast cancer.

The estimated numbers of new cases of female breast cancer at the global level were lower than those estimated with the GLOBOCAN 2020 (1). We estimated 1,977,212 new cases of female breast cancer in 2019, versus 2,261,419 new cases in GLOBOCAN data. But breast cancer resulted in 688,562 deaths in females globally, which is similar to GLOBOCAN data. Differences in estimation are due to different data sources and estimation methods. Breast cancer for females has surpassed lung cancer, ranking the first of cancer incidence worldwide in 2020 (1). We found that the absolute numbers of incidence cases, deaths, and DALYs increased globally between 1990 and 2019. Except that ASIR, ASMR and age-standardized DALY rates of breast cancer decreased globally at the same time. Much of this discrepancy may be driven by population growth and aging over the past three decades. Therefore, the declines in ASMR and age-standardized DALY rates have not necessarily resulted in a lower burden of breast cancer on the health systems. Based on our findings, breast cancer creates a huge socioeconomic burden of disease and disability worldwide, and the overall burden could continue to worsen unless risk factors such as alcohol use, high BMI, and high FPG are eliminated.

Since 1990, both the incidence case and ASIR of breast cancer generally increased with increasing SDI. Higher incidence of breast cancer in high SDI quintiles could be caused by the ageing population and the lifestyle choices that increase the exposure to risk factors; some of the risk factors for breast cancer are found to be more prevalent in high SDI quintiles than in low ones (12). With its extremely large population, East Asia, although having a lower ASIR than high-income regions, was found with the highest number of breast cancer incidence cases in 2019. From 1990 to 2019, the trends of breast cancer incidence in different regions and countries were inconsistent. Different from the pattern that ASIR increased throughout the study period in low SDI quintiles, the pattern in high SDI quintiles was that ASIR began to fall after 2000, which was largely attributed to the decline of menopausal hormone therapy and also possibly a plateau in cancer screening participation (13, 14). A previous randomized trial found that prior randomized use of conjugated equine estrogen plus medroxyprogesterone acetate, compared with placebo, among females who had an intact uterus, significantly increased the risk of breast cancer (15). Increased detection through widespread use of mammographic screening has been associated with the increased incidence of breast cancer worldwide (16, 17). The incidence of breast cancer increased in lower SDI quintiles, likely reflecting changes in the reproductive patterns coupled with the growing prevalence of obesity (18, 19). Furthermore, the incidence of breast cancer is anticipated to increase in the following years for the aggressive screenings and detections and longer life expectancy in lower SDI quintiles (20). An exceptionally high prevalence of BRCA mutation among Jewish women, in part accounts for the high incidence of breast cancer in Israel and certain European countries (21). The implementation of cancer registration results in better estimates of cancer burden. And cancer registration advancements may be another consideration for the increased trend observed in Sub-Saharan Africa (22). Thanks to the introduction of diagnostic services for screening patients at risk of cancer, more and more cases have been uncovered, which may also affect the findings presented in our study. In addition, our results revealed that EAPC was negatively associated with the baseline ASIR in 1990, meaning that some of the rapid increase occurred in areas where ASIR has been historically relatively low. This result reminds us that breast cancer should not be placed at a lower priority in disease prevention and treatment compared with other public health problem in those countries with low ASIR at baslin.

We found that the growth of the number of deaths and DALY decreased with the elevated SDI level. The ASMR and age-standardized DALY rates decreased in the high SDI and the high-middle SDI quintiles from 1990 to 2019, while slightly increased in other three quintiles. For example, although breast cancer along accounted for 30% of new cases for female cancer in United States of America (23), mortality of female breast cancer decreased from 1990 to 2019. The decrease in ASMR and age-standardized DALY rates may be due to an increase in survival resulting from improved management and treatment of breast cancer (23). An increasing number of females who are at an elevated risk of developing breast cancer choose prophylactic mastectomy to decrease their risk. It has been confirmed that prophylactic mastectomy could reduce the incidence and mortality of breast cancer by at least 81% (24). The ASMR and age-standardized DALY rates in Western Sub-Saharan Africa and Southern Sub-Saharan Africa African increased simultaneously. A previous study found that the 5-year age-standardized relative survival of breast cancer in Sub-Saharan Africa countries was significantly lower than in high-income countries (4). Without organized and population-based mammography screening programs, a lot of patients in Sub-Saharan Africa are less likely to catch breast cancer at a more treatable stage (25), and nearly a third of deaths could be prevented by early detection and adequate treatment (26). Therefore, clinical breast examination should be considered for breast cancer screening. A recent trial in Mumbai found that clinical breast examination conducted every two years significantly downstaged breast cancer at diagnosis and also led to a significant reduction of nearly 30% in mortality in females aged ≥50 (27). What’s more, we found that low-middle and low SDI experienced higher age-standardized DALYs. This result is partly magnified by the incidence of the breast cancer at younger ages, thus with a more severe impact on the life course. Therefore, we could speculate that the impact of breast cancer on productivity and family in low-middle and low SDI is enormous (28). A cohort in sub-Saharan Africa detects that the number of maternal orphans due to breast cancer exceeds the number of breast cancer deaths among females (29). To minimize the intergenerational effects of breast cancer deaths in sub-Saharan Africa, it is necessary to deliver adequate rehabilitation programs at crucial times in breast cancer survivors’ recovery. At the same time, support mechanisms are needed for affected families and children. Interestingly, China witnessed the highest number of breast cancer-related death in 2019, whereas the uptrend of mortality was more moderate than incidence. In China, the disparity in cancer mortality rate has proven to be far greater than incidence when urban and rural areas were compared. It can hardly be denied that medical resources were insufficient in rural area in the past few years. Nevertheless, a series of measures have be taken in China to reduce the care disparities between urban and rural areas, and these efforts may explain the sluggish increase in mortality of breast cancer. Greenland was found with a largest declining trend of ASIR and ASMR from 1990 to 2019. NORDCAN revealed that the incidence of breast cancer in Greenland was much lower than other Nordic countries while the mortality of breast cancer was approximately the same (30). Access to radiotherapy is lacking or absent in many Inuit regions, despite it being an essential component of cancer treatment (31). Therefore, breast cancer still imposes a considerable burden on health system in Greenland and other Inuit regions.

Globally, nearly 49% of breast cancer patients were aged 50–69 years. In well-resourced settings, WHO suggests organized, population-based mammography screening every 2 years for females aged 50 to 69 years (32). For females aged 70–75 years, the benefit to harm balance of mammography screening programmes is still uncertain. However, we noted that the proportion of the age 70+ years group increased globally and in most regions between 1990 and 2019. Therefore, there is an urgency for research in this area.

Our study showed an increasing contribution of high FPG and high BMI globally, and high FPG was recognized as the leading cause of global breast cancer deaths in 2019. High FPG and high BMI are associated with an increased likelihood of breast cancer (33, 34). High insulin resistance has particular negative consequences for cancer mortality for those postmenopausal females with normal weight (BMI < 25 kg/m^2^) who conventionally would be considered to be healthy compared to obese females (35). In particular, the association between BMI and breast cancer differs in the cancer subtypes and histopathologic features (34). The relationship between high BMI and breast cancer could be mediated by inflammatory foci which is attributable to macrophage infiltration of adipose tissue (35, 36). Inflammatory foci known as crownlike structures increases circulating pro-angiogenic factors which are closely related to an increased risk of breast cancer incidence and spread (35, 36). A recent study involving 10 cohorts in the United States of America, Australia, and Asia suggests that sustained weight loss, even modest amounts could reduce the risk of breast cancer for female aged 50 years and older (37).Government should try their best to provide a variety of measures to reverse the current epidemic of obesity and diabetes. Alcohol use was still the most important risk factors to breast cancer deaths in high quintiles, though the attributable death decreased from 1990 to 2019. A dose-response relationship between alcohol use and breast cancer has been found in previous study (38). However, the impact of light or moderate alcohol use on breast cancer remains to be established (39).The prevalence of alcohol use declined gobally over the last three decades, especially in the high SDI and the high-middle SDI quintiles (40). This may partly explain the the observed decrease in ASMR of breast cancer. A meta-analysis of 15 cohort studies found higher breast cancer risk for both current and former smoker comared to non-smoker (41). Smoking is associated with a slightly but significantly increased risk of breast cancer, particularly among females who started smoking at adolescent or peri-menarcheal ages (42). Females with a family history of breast cancer are at a greater risk of breast cancer when they smoke (42). Fortunately, significant reductions in the global prevalence of daily smoking are observed, which is matched with the finding of our study. We observed that low physical activity was also a contributor to breast cancer mortality. Recreational physical activity in adulthood also could attenuate the impact of family history or underlying genetic susceptibility on breast cancer risk for females (43). Different from previous studies by leveraging the GBD study (5, 6), we further analyzed the contribution of diet high in red meat to breast cancer deaths. It has been observed that association between red meat and breast cancer risk was of different magnitude in African-American and Caucasian (44). A low-fat dietary pattern that included increased vegetable, fruit, and grain consumption significantly reduces the risk of death as a result of breast cancer (45). The increased contribution of diet high in red meat to breast cancer deaths in lower SDI quintiles may result from the westernization of life style. Another study confirms that an overall healthy lifestyle may lower breast cancer risk among females with genetic risk factors of breast cancer (46). Therefore, it is essential to emphasize the necessity of healthy life style to the public to minimize breast cancer’s burden.

Two similar studies have estimated the burden of breast cancer based on the GBD study 2017 (5, 6). Although the methodology and the outputs are almost superimposable, there exist some differences of our study to previous studies. GBD 2019 provided the most up-to-date assessment of the descriptive epidemiology of breast cancer for 204 countries and territories from 1990 to 2019. And the overall burden of breast cancer continued to worsen in 2019. Moreover, we firstly investigated the burden of breast cancer linked to diet high in red meat, but the contribution ratio of diet high in red meat was relatively weak. In addition, compared to the study by Ji, P et al, the relationship between SDI and ASIR from 1990 to 2019 for global and 21 GBD regions was demonstrated by our study.

Our study has limitations. Firstly, our results depended on the quality and quantity of data used in the modeling. The paucity of rigorous epidemiological data and unusable data in several countries could affect the produced estimates. Secondly, we did not further assess the burden and trends of cancer subtypes due to the lack of histopathologic data. Thirdly, it will be more convincing if we get a comparative assessment on other risk factors. Unfortunately, the data about other risk factors were not available on the online dataset. Lastly, SDI is not a metric agreed by governments, and never subjected to global consultations, and somewhat affected by assumptions for the lack of good country-level data. therefore, the results should be interpreted with caution.

In conclusion, breast cancer remained a major public health burden globally. Over the past three decades, the numbers of incidence, death and DALY increased globally, but the ASMR and age-standardized DALY rates decreased in the high SDI and the high-middle SDI quintiles. Meanwhile, the burden rapidly increased in lower SDI quintiles. Besides, the proportion of the 70+ years age group increased globally. With more countries facing an aging population, it is important to be aware of the potentially increased burden of breast cancer. Moreover, the risk factors varied between different regions. These considerations should provide guidance for the development of control strategies of breast cancer, and more appropriate interventions are targeted based on local characteristics.

## Data Availability Statement

Publicly available datasets were analyzed in this study. This data can be found here: http://ghdx.healthdata.org/gbd-results-tool.

## Ethics Statement

Ethical review and approval was not required for the study on human participants in accordance with the local legislation and institutional requirements. Written informed consent from the participants’ legal guardian/next of kin was not required to participate in this study in accordance with the national legislation and the institutional requirements.

## Author Contributions

SX, YL, and ZL contributed to conception and design of the study. SX and ZL organized the database. SX, TZ and WL performed the statistical analysis. JiZ, JC and JuZ wrote the first draft of the manuscript. YaC, YX, YeC, and ZL wrote sections of the manuscript. All authors contributed to the article and approved the submitted version.

## Funding

This work was supported by the Medical Scientific Research Foundation of Guangdong Province, China (grant numbers A2021432, B2021448), Shantou Medical Science and Technology Planning Project (grant number 200622115260639), Undergraduate Innovation and Entrepreneurship Training Program Project of Shantou University (grant number 201910560249), and the Bethune-Ethicon Excellence Surgery Fund Project (grant number HZB-20181119-25).

## Conflict of Interest

The authors declare that the research was conducted in the absence of any commercial or financial relationships that could be construed as a potential conflict of interest.
