# Supplementary figures and images for: The Global, Regional, and National Burden and Trends of Breast Cancer From 1990 to 2019: Results From the Global Burden of Disease Study 2019
Source: Front Oncol. 2021 May 21;11:689562. doi: 10.3389/fonc.2021.689562 (PMC8176863; doi:10.3389/fonc.2021.689562)

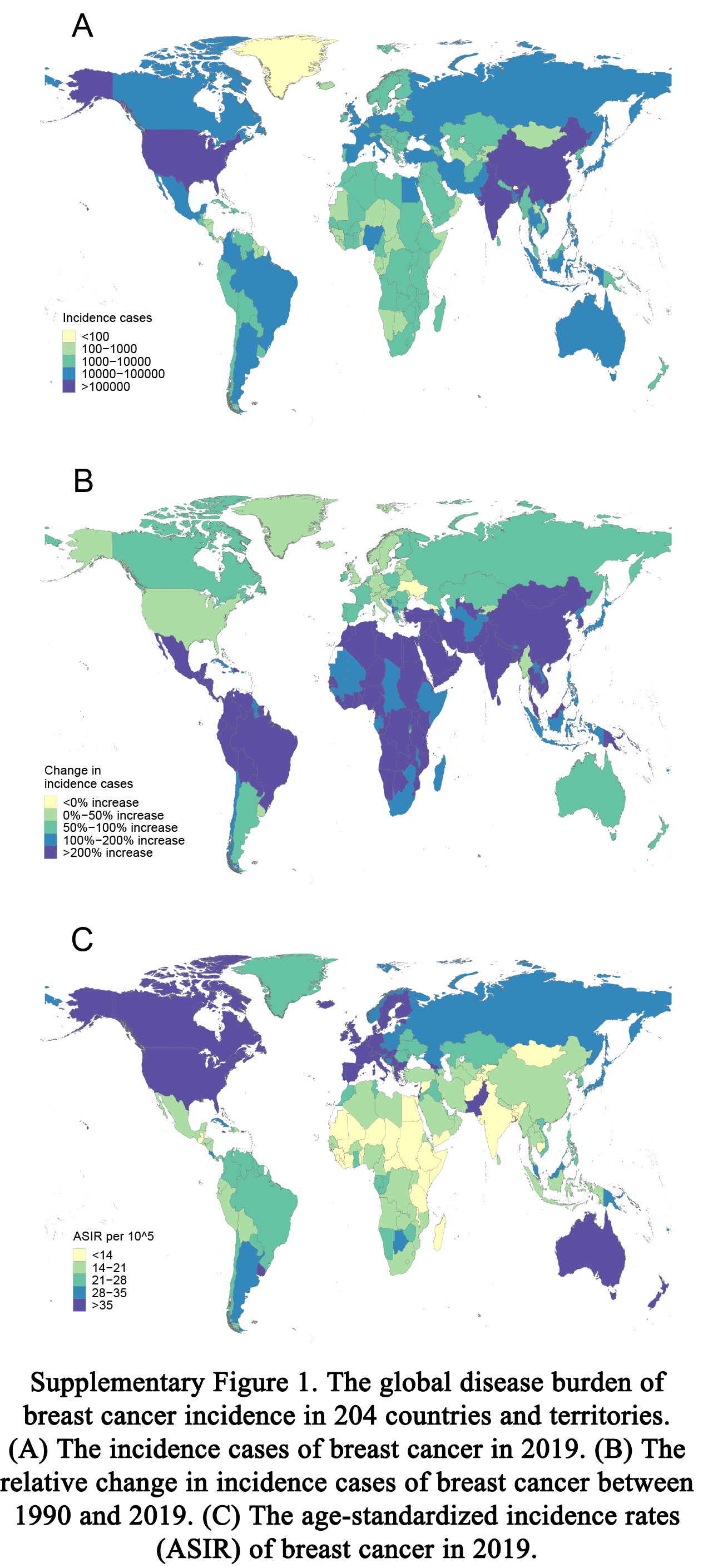

Supplement: Supplementary file 1 [file Image_1.tif]

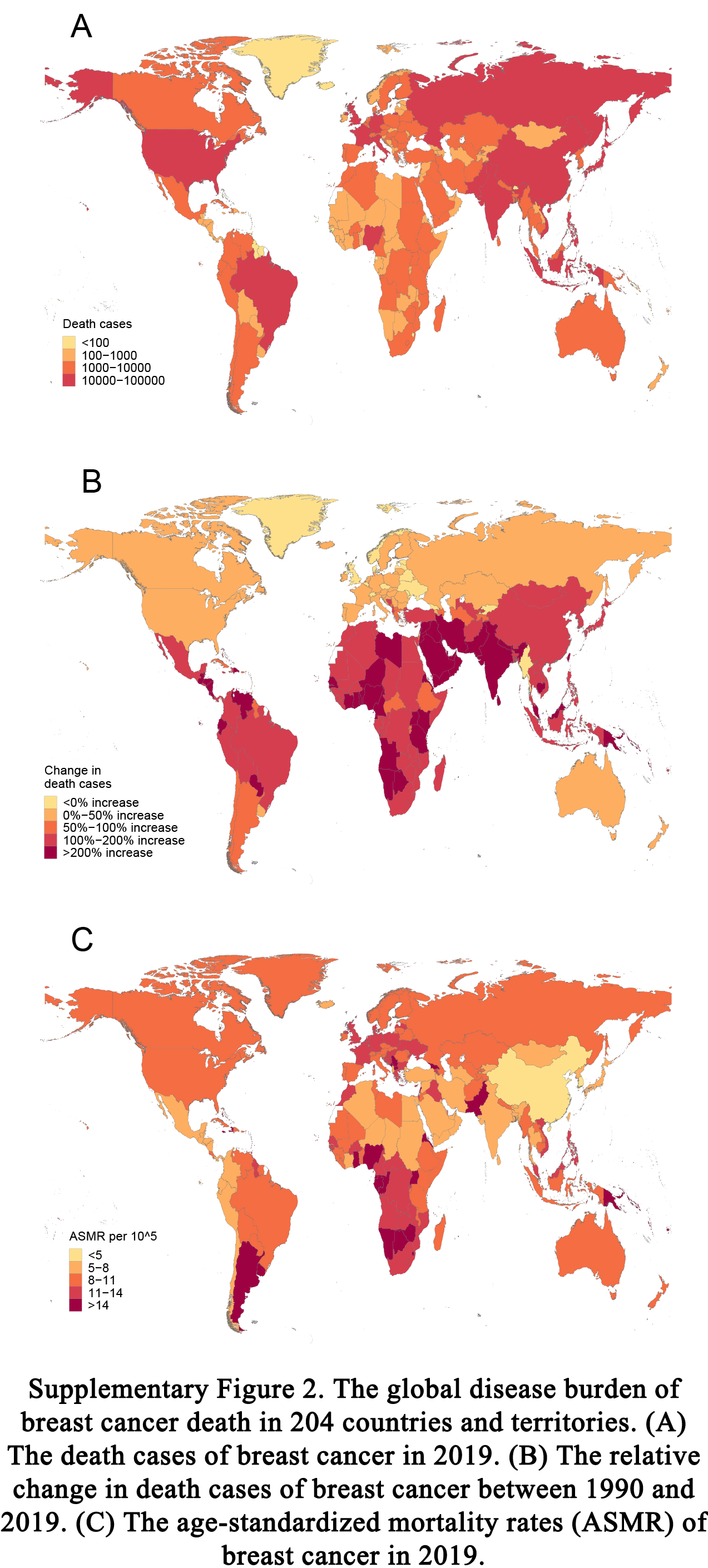

Supplement: Supplementary file 2 [file Image_2.tif]

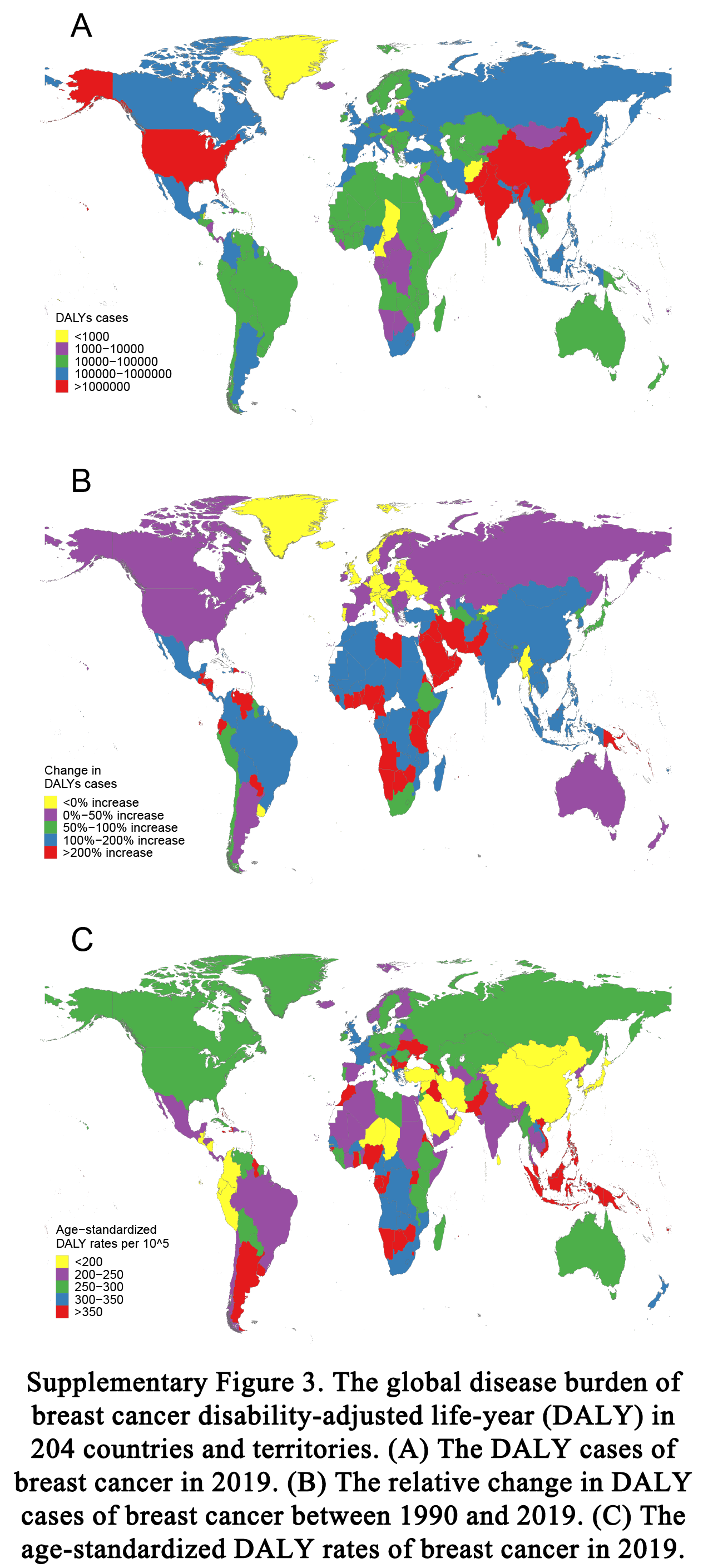

Supplement: Supplementary file 3 [file Image_3.tif]

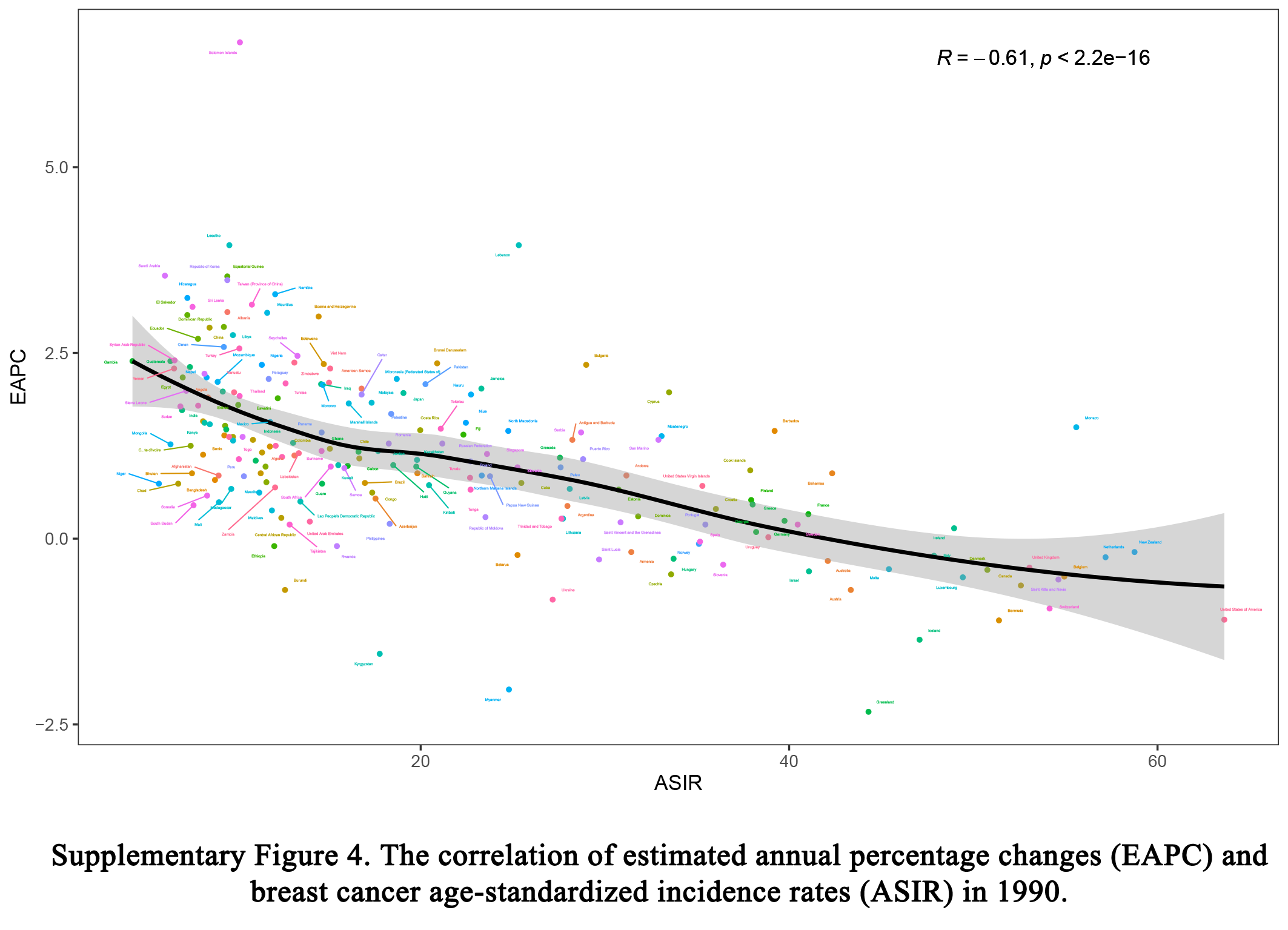

Supplement: Supplementary file 4 [file Image_4.tif]
