# Supplementary material for: The Global, Regional, and National Burden and Trends of Breast Cancer From 1990 to 2019: Results From the Global Burden of Disease Study 2019
Source: Front Oncol. 2021 May 21;11:689562. doi: 10.3389/fonc.2021.689562 (PMC8176863; doi:10.3389/fonc.2021.689562)
Supplement: Supplementary file 5 [file Table_1.docx]

**Supplementary Table 1. Breast cancer incidence cases, age-standardized incidence rate, deaths, age-standardized mortality rate, DALYs, and age-standardized DALY rates in 2019.**

| **Characteristics** | **Incidence cases (95%UI)** | **ASIR per 10^5^ (95% UI)** | **Deaths (95% UI)** | **ASMR per 10^5^ (95% UI)** | **DALYs (95% UI)** | **Age-standardized DALY rates per 10^5^ (95% UI)** |
| --- | --- | --- | --- | --- | --- | --- |
| Afghanistan | 1988  (1486-2606) | 11.61  (8.79-15.07) | 1311  (987-1705) | 8.67  (6.59-11.16) | 49211  (36828-65071) | 262.25  (198.13-342.83) |
| Albania | 764  (555-1031) | 20.73  (15.07-28.00) | 258  (190-343) | 6.50  (4.83-8.68) | 7379  (5418-9965) | 199.56  (145.58-271.40) |
| Algeria | 7052  (5307-8987) | 18.08  (13.79-22.73) | 2662  (2013-3352) | 7.58  (5.83-9.50) | 89155  (67273-114020) | 224.38  (170.32-285.40) |
| American Samoa | 14  (11-18) | 29.00  (23.05-36.41) | 8  (6-10) | 16.81  (13.50-20.65) | 234  (185-294) | 458.91  (363.97-575.16) |
| Andorra | 55  (39-74) | 40.46  (28.71-54.12) | 13  (9-17) | 9.02  (6.48-11.97) | 348  (250-473) | 254.46  (183.10-345.10) |
| Angola | 1978  (1408-2660) | 14.88  (11.10-19.68) | 1338  (967-1790) | 11.48  (8.78-15.03) | 45997  (32317-62266) | 310.10  (224.17-414.75) |
| Antigua and Barbuda | 45  (38-54) | 42.79  (36.40-50.27) | 16  (14-19) | 16.44  (14.25-18.90) | 462  (391-547) | 430.14  (366.22-506.44) |
| Argentina | 17868  (13834-22719) | 34.29  (26.50-43.74) | 8476  (7818-9135) | 15.84  (14.65-17.07) | 209653  (194978-225664) | 406.21  (377.09-437.31) |
| Armenia | 1363  (1116-1641) | 34.18  (27.86-41.23) | 558  (465-662) | 13.75  (11.43-16.36) | 15646  (12961-18825) | 392.55  (321.77-472.39) |
| Australia | 15656  (12089-20129) | 42.61  (32.85-55.11) | 3711  (3324-4031) | 9.15  (8.31-9.87) | 93004  (85086-102208) | 256.54  (235.53-281.65) |
| Austria | 5698  (4584-6999) | 37.09  (29.80-45.64) | 1776  (1598-1927) | 9.82  (8.95-10.56) | 38632  (35426-41911) | 250.76  (230.47-271.27) |
| Azerbaijan | 2300  (1834-2862) | 20.76  (16.63-25.66) | 993  (792-1234) | 9.84  (7.87-12.11) | 33531  (26534-41824) | 295.10  (235.93-364.24) |
| Bahamas | 211  (169-263) | 49.88  (40.22-61.96) | 80  (65-99) | 19.98  (16.44-24.62) | 2524  (2015-3160) | 586.74  (473.21-732.64) |
| Bahrain | 349  (273-437) | 26.16  (21.08-31.89) | 104  (83-128) | 10.61  (8.68-12.83) | 3625  (2883-4525) | 253.45  (203.02-311.01) |
| Bangladesh | 17812  (13804-22827) | 12.49  (9.74-15.90) | 9884  (7761-12395) | 7.20  (5.69-8.97) | 327880  (254782-416587) | 225.98  (176.27-286.64) |
| Barbados | 254  (206-305) | 55.46  (44.89-66.62) | 95  (78-112) | 20.04  (16.44-23.69) | 2431  (1964-2905) | 540.70  (434.09-649.36) |
| Belarus | 4133  (3140-5455) | 27.96  (21.19-36.97) | 1297  (1006-1668) | 8.40  (6.48-10.76) | 36206  (27659-47348) | 246.24  (187.48-324.37) |
| Belgium | 9697  (7576-12408) | 49.86  (38.74-64.47) | 2919  (2586-3184) | 12.69  (11.51-13.72) | 63769  (58318-70252) | 329.56  (302.91-362.10) |
| Belize | 44  (37-53) | 13.82  (11.63-16.32) | 18  (16-22) | 6.18  (5.26-7.26) | 612  (512-726) | 184.09  (154.46-217.69) |
| Benin | 638  (452-874) | 11.32  (8.25-15.07) | 440  (319-590) | 8.57  (6.39-11.12) | 14381  (10041-20040) | 235.09  (168.67-319.41) |
| Bermuda | 50  (41-63) | 43.07  (34.78-54.36) | 14  (11-18) | 10.99  (8.92-13.68) | 314  (257-393) | 275.46  (224.82-346.91) |
| Bhutan | 63  (44-87) | 10.20  (7.21-13.94) | 35  (24-47) | 5.95  (4.27-8.06) | 1081  (733-1511) | 169.55  (116.68-233.73) |
| Bolivia  (Plurinational State of) | 1704  (1282-2278) | 18.08  (13.73-23.92) | 902  (689-1167) | 10.09  (7.88-12.92) | 27086  (19973-35998) | 279.81  (208.91-367.76) |
| Bosnia and Herzegovina | 1635  (1253-2105) | 29.89  (22.89-38.70) | 668  (523-838) | 11.70  (9.22-14.72) | 16951  (13093-21750) | 310.53  (240.59-400.99) |
| Botswana | 448  (283-669) | 28.22  (18.51-41.11) | 235  (151-342) | 17.27  (11.63-24.48) | 7638  (4706-11389) | 448.82  (288.53-656.16) |
| Brazil | 52473  (49142-55904) | 21.51  (20.11-22.91) | 20121  (18758-21373) | 8.43  (7.83-8.97) | 598059  (563114-636739) | 243.19  (229.06-258.82) |
| Brunei Darussalam | 142  (115-175) | 34.70  (28.77-41.68) | 43  (36-52) | 12.38  (10.52-14.45) | 1599  (1312-1935) | 374.62  (312.65-447.02) |
| Bulgaria | 5122  (4003-6482) | 41.89  (32.36-53.33) | 1710  (1364-2121) | 12.86  (10.18-16.04) | 44163  (34651-55947) | 371.05  (287.51-473.28) |
| Burkina Faso | 1575  (1203-2013) | 14.96  (11.81-18.54) | 1088  (854-1366) | 11.48  (9.23-14.04) | 35827  (26850-46134) | 310.67  (240.82-391.73) |
| Burundi | 600  (413-847) | 11.33  (8.01-15.69) | 436  (303-611) | 9.19  (6.61-12.75) | 14640  (9996-20722) | 248.52  (173.72-348.39) |
| Cabo Verde | 69  (56-85) | 15.21  (12.16-18.72) | 38  (30-47) | 8.80  (6.93-10.75) | 1009  (811-1235) | 216.64  (173.77-264.00) |
| Cambodia | 1816  (1374-2290) | 13.30  (10.20-16.50) | 1029  (794-1283) | 8.07  (6.29-9.87) | 34880  (26336-44358) | 247.73  (188.91-311.11) |
| Cameroon | 2256  (1523-3301) | 16.06  (11.09-22.93) | 1462  (1004-2098) | 11.63  (8.32-16.21) | 48818  (32371-72033) | 318.74  (218.36-464.22) |
| Canada | 28616  (22312-36219) | 47.20  (36.83-60.51) | 6565  (5890-7168) | 9.76  (8.86-10.60) | 159852  (144867-175827) | 268.16  (244.44-294.16) |
| Central African Republic | 339  (212-547) | 13.35  (8.60-21.58) | 267  (169-429) | 11.69  (7.60-18.57) | 9354  (5800-15283) | 324.31  (206.39-520.37) |
| Chad | 555  (384-752) | 8.40  (5.93-11.20) | 406  (285-544) | 6.66  (4.78-8.81) | 13785  (9380-18992) | 192.45  (134.08-260.53) |
| Chile | 4937  (3837-6318) | 21.19  (16.42-27.07) | 1831  (1667-2003) | 7.74  (7.03-8.45) | 47034  (42569-51588) | 202.16  (183.47-221.80) |
| China | 375484  (296626-469983) | 18.32  (14.50-22.93) | 96306  (77323-118090) | 4.85  (3.91-5.92) | 2957454  (2408511-3590166) | 144.15  (117.26-174.99) |
| Colombia | 11162  (8522-14373) | 21.21  (16.19-27.31) | 3401  (2645-4331) | 6.38  (4.98-8.15) | 102053  (78322-132897) | 193.84  (148.98-252.18) |
| Comoros | 78  (57-103) | 15.06  (11.32-19.67) | 56  (43-73) | 11.55  (8.89-14.91) | 1680  (1208-2237) | 308.00  (225.31-409.92) |
| Congo | 661  (392-989) | 21.24  (13.00-31.00) | 441  (268-653) | 16.05  (10.04-23.06) | 14893  (8715-22497) | 433.19  (264.14-644.13) |
| Cook Islands | 11  (8-14) | 47.41  (35.02-60.84) | 5  (4-6) | 20.14  (15.93-24.93) | 122  (93-156) | 538.49  (401.42-692.50) |
| Costa Rica | 1514  (1159-1926) | 29.12  (22.37-36.96) | 438  (345-551) | 8.45  (6.64-10.62) | 12157  (9401-15609) | 232.64  (179.96-298.70) |
| Croatia | 2865  (2214-3646) | 37.34  (28.62-47.88) | 1001  (785-1234) | 11.60  (9.08-14.35) | 21804  (16915-27534) | 284.42  (219.29-361.55) |
| Cuba | 5590  (4479-6915) | 31.13  (24.96-38.59) | 1776  (1460-2163) | 9.41  (7.74-11.47) | 44777  (35928-55471) | 252.31  (202.18-311.56) |
| Cyprus | 985  (816-1185) | 53.77  (44.57-64.54) | 221  (191-252) | 12.17  (10.56-13.86) | 5757  (4911-6733) | 315.18  (268.23-368.19) |
| Czechia | 5748  (4615-7085) | 30.97  (24.80-38.29) | 1904  (1556-2285) | 9.23  (7.55-11.14) | 42582  (34701-51934) | 228.24  (184.72-279.49) |
| Côte d'Ivoire | 1323  (935-1799) | 10.35  (7.71-13.64) | 900  (656-1197) | 7.96  (6.08-10.20) | 30587  (21496-41441) | 214.08  (156.01-283.97) |
| Democratic People's Republic of Korea | 4718  (3329-6521) | 14.15  (10.06-19.62) | 2384  (1761-3161) | 7.28  (5.45-9.63) | 75655  (53147-103855) | 225.05  (158.46-310.22) |
| Democratic Republic of the Congo | 6528  (4644-8824) | 16.02  (11.35-21.78) | 4704  (3342-6355) | 12.94  (9.16-17.47) | 155532  (109555-207861) | 342.87  (244.38-462.04) |
| Denmark | 4290  (3313-5449) | 43.68  (33.52-56.10) | 1399  (1243-1543) | 12.32  (11.02-13.49) | 30451  (27299-33847) | 306.57  (275.75-339.80) |
| Djibouti | 100  (66-145) | 14.19  (10.16-19.36) | 65  (44-92) | 10.70  (8.07-14.30) | 2184  (1389-3216) | 275.22  (188.54-385.54) |
| Dominica | 28  (23-35) | 32.96  (26.34-41.45) | 14  (12-17) | 16.11  (13.17-19.85) | 362  (287-457) | 431.47  (340.98-547.52) |
| Dominican Republic | 1850  (1321-2499) | 18.63  (13.41-25.00) | 798  (590-1044) | 8.40  (6.27-10.94) | 25037  (17808-33618) | 247.58  (177.49-330.69) |
| Ecuador | 2553  (1973-3323) | 16.16  (12.53-20.99) | 1044  (818-1346) | 6.90  (5.43-8.83) | 31654  (24201-41342) | 197.62  (151.90-257.13) |
| Egypt | 10637  (7384-14572) | 13.62  (9.36-18.58) | 4672  (3173-6369) | 6.44  (4.37-8.72) | 163742  (113133-224191) | 204.66  (140.41-278.82) |
| El Salvador | 1020  (738-1375) | 17.38  (12.58-23.47) | 373  (278-489) | 6.27  (4.66-8.23) | 11116  (8050-14929) | 189.62  (137.65-254.43) |
| Equatorial Guinea | 127  (70-212) | 22.77  (13.11-36.94) | 77  (43-126) | 15.99  (9.45-25.42) | 2446  (1329-4158) | 400.88  (222.66-663.26) |
| Eritrea | 534  (380-750) | 17.22  (12.64-23.56) | 385  (279-536) | 14.05  (10.45-19.07) | 13027  (9108-18510) | 372.60  (270.65-515.04) |
| Estonia | 824  (635-1047) | 36.89  (28.23-47.08) | 260  (205-327) | 10.29  (8.06-12.96) | 6126  (4767-7824) | 279.70  (213.91-359.69) |
| Eswatini | 119  (72-180) | 19.47  (12.33-28.54) | 80  (50-120) | 14.82  (9.57-21.12) | 2460  (1442-3822) | 368.29  (223.88-555.24) |
| Ethiopia | 5900  (4638-7416) | 12.54  (10.08-15.28) | 4105  (3298-4955) | 9.71  (7.96-11.64) | 129579  (101092-161688) | 251.93  (201.58-306.67) |
| Fiji | 251  (185-332) | 31.15  (23.41-40.88) | 147  (109-193) | 20.26  (15.47-26.22) | 4707  (3459-6193) | 556.10  (415.83-722.92) |
| Finland | 4391  (3379-5570) | 44.09  (33.70-56.80) | 1046  (931-1151) | 8.86  (8.02-9.67) | 23955  (21524-26714) | 242.41  (218.26-269.78) |
| France | 51276  (39714-66118) | 46.35  (35.44-60.21) | 15326  (13075-16868) | 11.16  (9.95-12.10) | 332727  (298541-365857) | 302.13  (274.48-332.24) |
| Gabon | 251  (162-356) | 22.18  (14.41-30.73) | 161  (105-224) | 16.00  (10.48-21.79) | 4834  (3107-6896) | 396.44  (259.21-557.30) |
| Gambia | 96  (62-136) | 8.87  (5.79-12.59) | 65  (43-92) | 6.44  (4.25-9.05) | 2101  (1356-3025) | 183.03  (118.73-262.00) |
| Georgia | 1931  (1591-2332) | 36.42  (29.97-43.92) | 905  (755-1079) | 16.00  (13.17-19.08) | 24976  (20575-29962) | 476.59  (391.61-571.38) |
| Germany | 70173  (53859-90348) | 44.25  (33.94-57.97) | 21652  (19398-23510) | 11.53  (10.50-12.37) | 470731  (428737-511632) | 299.69  (274.96-326.05) |
| Ghana | 4702  (3597-6064) | 25.09  (19.70-31.93) | 2902  (2258-3703) | 17.21  (13.71-21.66) | 95504  (72117-124147) | 475.30  (367.15-609.91) |
| Greece | 8800  (6819-11260) | 45.26  (34.59-58.80) | 2822  (2516-3080) | 11.74  (10.67-12.67) | 59388  (54309-65320) | 308.69  (283.45-337.63) |
| Greenland | 16  (12-20) | 21.94  (17.27-27.70) | 7  (5-8) | 9.84  (7.79-12.25) | 198  (151-255) | 269.69  (209.94-343.95) |
| Grenada | 42  (36-47) | 36.73  (31.96-41.53) | 18  (16-20) | 16.46  (14.58-18.37) | 506  (441-578) | 438.70  (383.30-499.92) |
| Guam | 35  (29-43) | 19.09  (15.56-23.40) | 17  (14-20) | 9.09  (7.53-11.06) | 477  (389-585) | 256.18  (210.13-312.48) |
| Guatemala | 1453  (1111-1881) | 12.24  (9.46-15.81) | 669  (527-848) | 5.96  (4.73-7.51) | 20825  (16036-26673) | 170.11  (132.03-218.01) |
| Guinea | 780  (562-1054) | 12.67  (9.28-16.78) | 575  (419-760) | 9.89  (7.30-12.95) | 18603  (13383-25427) | 284.91  (206.56-385.22) |
| Guinea-Bissau | 144  (97-211) | 16.14  (11.23-23.32) | 100  (69-145) | 12.42  (8.80-17.91) | 3533  (2368-5289) | 360.72  (247.94-524.34) |
| Guyana | 181  (135-235) | 26.14  (19.82-33.65) | 88  (67-113) | 13.58  (10.60-17.20) | 2878  (2141-3761) | 403.17  (303.29-525.56) |
| Haiti | 1962  (1246-2838) | 23.28  (15.02-33.22) | 1214  (777-1746) | 15.49  (10.16-21.81) | 42709  (27127-63472) | 474.39  (303.44-690.22) |
| Honduras | 957  (670-1326) | 14.51  (10.44-20.04) | 442  (324-600) | 7.17  (5.41-9.51) | 13781  (9676-19199) | 202.18  (145.25-279.24) |
| Hungary | 5974  (4874-7315) | 35.38  (28.38-43.51) | 2207  (1813-2651) | 11.78  (9.61-14.19) | 51732  (42000-62880) | 307.30  (248.82-376.39) |
| Iceland | 173  (145-203) | 35.23  (29.74-41.05) | 43  (36-49) | 7.73  (6.65-8.75) | 1055  (921-1186) | 216.43  (189.08-242.70) |
| India | 146090  (112452-183482) | 11.81  (9.15-14.82) | 83510  (64550-105994) | 7.12  (5.53-9.01) | 2697350  (2090173-3424541) | 211.95  (164.23-268.62) |
| Indonesia | 50985  (39200-66927) | 19.14  (14.88-24.71) | 26420  (20392-33925) | 10.69  (8.36-13.46) | 980859  (763277-1264306) | 358.03  (279.53-457.55) |
| Iran  (Islamic Republic of) | 14863  (13372-16590) | 17.10  (15.41-19.12) | 4760  (4362-5250) | 5.99  (5.46-6.61) | 163091  (148806-179221) | 185.58  (169.69-203.38) |
| Iraq | 7902  (5795-10587) | 26.20  (19.67-34.67) | 3015  (2252-3985) | 11.10  (8.46-14.35) | 110411  (80899-149282) | 359.46  (267.41-478.92) |
| Ireland | 3408  (2616-4345) | 49.21  (37.41-62.77) | 804  (724-886) | 10.98  (9.94-12.05) | 20595  (18570-22919) | 298.01  (269.14-332.53) |
| Israel | 4464  (3441-5702) | 41.82  (32.02-53.45) | 1361  (1231-1476) | 11.81  (10.75-12.73) | 32853  (30058-35655) | 310.00  (284.49-336.96) |
| Italy | 53565  (41369-68124) | 47.15  (36.37-60.52) | 14450  (12591-15499) | 10.25  (9.24-10.89) | 320926  (294648-346641) | 281.14  (260.61-302.81) |
| Jamaica | 1224  (942-1553) | 40.93  (31.49-52.07) | 472  (372-582) | 15.46  (12.18-19.13) | 14062  (10953-17865) | 470.54  (366.02-597.65) |
| Japan | 74597  (59578-91312) | 31.02  (24.85-38.16) | 16038  (13489-17428) | 5.37  (4.85-5.72) | 397774  (358070-434855) | 172.96  (160.51-187.61) |
| Jordan | 2075  (1591-2658) | 24.95  (19.32-31.54) | 685  (536-869) | 9.51  (7.51-11.86) | 23477  (18164-30109) | 274.97  (213.96-348.47) |
| Kazakhstan | 4252  (3558-5002) | 22.66  (19.06-26.53) | 1686  (1444-1956) | 9.64  (8.28-11.13) | 51889  (43913-61260) | 273.27  (231.39-319.63) |
| Kenya | 3398  (2485-4509) | 13.33  (9.96-17.25) | 2321  (1747-3132) | 10.29  (7.91-13.40) | 75590  (55661-104567) | 270.92  (203.31-365.82) |
| Kiribati | 21  (15-28) | 26.01  (19.05-35.71) | 14  (10-20) | 20.08  (14.94-27.44) | 500  (352-686) | 577.12  (415.00-790.43) |
| Kuwait | 724  (570-930) | 18.11  (14.60-23.16) | 171  (138-218) | 5.46  (4.46-6.92) | 6148  (4902-7991) | 152.52  (124.02-195.61) |
| Kyrgyzstan | 651  (552-764) | 12.46  (10.61-14.54) | 295  (254-342) | 6.20  (5.38-7.15) | 9358  (7981-10980) | 175.17  (150.77-204.48) |
| Lao People's Democratic Republic | 859  (605-1227) | 16.18  (11.64-22.85) | 535  (384-760) | 10.93  (8.01-15.22) | 18600  (13034-26869) | 334.56  (238.80-478.36) |
| Latvia | 1098  (840-1428) | 32.10  (24.39-42.26) | 422  (324-541) | 11.19  (8.49-14.44) | 10114  (7692-13134) | 307.47  (229.23-405.99) |
| Lebanon | 3553  (2680-4666) | 67.42  (50.69-88.46) | 1028  (794-1344) | 19.79  (15.23-25.84) | 30918  (23510-40738) | 587.93  (446.26-774.74) |
| Lesotho | 294  (182-445) | 22.18  (14.24-33.09) | 214  (136-320) | 17.67  (11.57-25.60) | 6506  (3913-10029) | 456.23  (283.94-690.25) |
| Liberia | 312  (210-473) | 12.43  (8.57-18.74) | 208  (142-315) | 9.22  (6.51-13.72) | 7047  (4696-10723) | 256.27  (173.06-388.07) |
| Libya | 1365  (961-1906) | 20.48  (14.58-27.95) | 517  (375-707) | 8.63  (6.34-11.52) | 18488  (13162-25563) | 272.37  (195.87-371.83) |
| Lithuania | 1367  (1101-1679) | 28.69  (23.06-35.47) | 537  (437-655) | 9.96  (8.04-12.20) | 13215  (10628-16387) | 281.15  (223.73-351.15) |
| Luxembourg | 382  (308-474) | 40.94  (32.99-50.68) | 106  (90-124) | 10.40  (8.87-12.15) | 2507  (2157-2912) | 268.93  (230.92-312.40) |
| Madagascar | 1629  (1145-2213) | 12.15  (8.86-16.23) | 1145  (821-1544) | 9.56  (7.13-12.55) | 40285  (28384-54797) | 269.73  (194.31-362.80) |
| Malawi | 1061  (784-1370) | 13.52  (10.27-17.07) | 774  (584-983) | 10.97  (8.54-13.56) | 23578  (16926-31098) | 272.27  (201.52-349.96) |
| Malaysia | 8663  (6567-10970) | 29.44  (22.38-37.22) | 3530  (2724-4462) | 12.93  (9.96-16.25) | 111532  (85615-140610) | 374.38  (287.82-470.43) |
| Maldives | 56  (44-68) | 14.68  (11.73-17.80) | 20  (16-24) | 6.18  (5.03-7.48) | 625  (498-766) | 162.36  (130.87-198.44) |
| Mali | 1131  (824-1497) | 11.32  (8.45-14.80) | 777  (581-1014) | 8.25  (6.29-10.73) | 26016  (18723-34415) | 246.84  (181.42-323.14) |
| Malta | 318  (262-384) | 41.59  (34.27-50.39) | 96  (82-111) | 11.07  (9.57-12.77) | 2273  (1962-2644) | 303.02  (263.62-350.39) |
| Marshall Islands | 12  (8-19) | 28.59  (18.94-42.55) | 7  (5-11) | 19.35  (13.27-28.22) | 274  (175-413) | 591.31  (389.43-883.73) |
| Mauritania | 303  (213-406) | 13.34  (9.41-17.76) | 193  (137-255) | 9.09  (6.60-11.86) | 5897  (4075-7919) | 247.92  (171.82-332.34) |
| Mauritius | 482  (382-601) | 27.91  (22.15-34.57) | 205  (165-252) | 11.86  (9.55-14.54) | 6133  (4868-7615) | 352.45  (279.72-437.32) |
| Mexico | 24442  (19918-29949) | 19.62  (16.03-23.99) | 8097  (6718-9852) | 6.72  (5.59-8.14) | 256474  (212636-312818) | 203.09  (168.58-247.33) |
| Micronesia  (Federated States of) | 28  (16-46) | 34.73  (21.10-56.09) | 16  (10-26) | 22.82  (14.73-35.65) | 540  (299-901) | 641.84  (379.58-1051.06) |
| Monaco | 58  (42-73) | 78.51  (57.30-101.60) | 16  (12-19) | 17.37  (12.87-21.73) | 354  (263-447) | 485.66  (357.47-631.23) |
| Mongolia | 283  (201-387) | 9.92  (7.21-13.25) | 141  (101-191) | 5.73  (4.29-7.56) | 4825  (3414-6614) | 160.54  (116.18-217.58) |
| Montenegro | 413  (329-508) | 45.73  (36.32-56.20) | 136  (111-164) | 14.56  (11.89-17.57) | 3711  (2965-4557) | 410.37  (327.88-505.24) |
| Morocco | 9893  (7152-13669) | 26.85  (19.54-36.75) | 4464  (3256-6065) | 12.60  (9.31-16.92) | 160775  (116654-222518) | 430.23  (312.86-589.38) |
| Mozambique | 1904  (1272-2680) | 15.40  (10.90-21.33) | 1374  (955-1911) | 12.53  (9.13-17.04) | 44978  (29266-64027) | 325.30  (222.04-457.53) |
| Myanmar | 7623  (6052-9658) | 14.92  (12.04-18.75) | 4617  (3781-5787) | 9.59  (7.96-11.88) | 147752  (116978-188773) | 281.10  (225.30-356.13) |
| Namibia | 436  (299-638) | 28.00  (19.55-40.23) | 259  (181-371) | 17.83  (12.82-24.79) | 8061  (5341-11992) | 496.94  (339.01-728.95) |
| Nauru | 2  (2-4) | 39.88  (26.59-54.70) | 1  (1-2) | 21.75  (15.22-29.04) | 43  (27-61) | 654.56  (434.39-904.40) |
| Nepal | 3716  (2723-4973) | 15.32  (11.26-20.42) | 2254  (1673-3002) | 9.63  (7.15-12.69) | 72369  (52531-97254) | 292.73  (213.88-392.33) |
| Netherlands | 16707  (12927-21329) | 57.76  (44.53-74.28) | 4350  (3894-4765) | 13.15  (11.94-14.29) | 100294  (91017-110124) | 347.09  (316.76-380.81) |
| New Zealand | 3613  (2795-4659) | 53.49  (41.12-69.13) | 769  (696-832) | 10.49  (9.62-11.29) | 20902  (19103-22926) | 317.85  (291.18-347.10) |
| Nicaragua | 875  (698-1106) | 18.03  (14.56-22.47) | 302  (248-371) | 6.77  (5.64-8.11) | 9204  (7295-11671) | 183.82  (147.40-230.62) |
| Niger | 671  (446-1012) | 7.49  (5.12-11.13) | 479  (324-713) | 5.83  (4.04-8.48) | 15959  (10594-24086) | 164.30  (111.09-245.53) |
| Nigeria | 21190  (14416-29569) | 20.64  (14.35-28.16) | 13385  (9417-18494) | 14.29  (10.31-19.24) | 436920  (303424-621718) | 398.46  (281.23-550.37) |
| Niue | 1  (0-1) | 35.51  (23.21-48.79) | 0  (0-0) | 16.47  (10.98-21.88) | 9  (6-13) | 454.18  (297.53-623.51) |
| North Macedonia | 1168  (882-1513) | 37.54  (28.35-48.78) | 430  (335-542) | 14.08  (11.04-17.62) | 11925  (9034-15302) | 382.72  (291.08-487.98) |
| Northern Mariana Islands | 18  (14-23) | 32.08  (25.22-40.40) | 7  (5-9) | 13.90  (11.28-17.22) | 215  (167-275) | 376.51  (298.97-475.00) |
| Norway | 2927  (2351-3688) | 34.97  (27.88-44.17) | 797  (706-871) | 8.31  (7.52-9.02) | 18341  (16769-20127) | 219.07  (201.47-240.57) |
| Oman | 448  (365-534) | 19.47  (16.31-22.93) | 133  (111-158) | 7.65  (6.48-9.03) | 4504  (3688-5378) | 187.99  (157.76-222.14) |
| Pakistan | 51438  (37937-69293) | 38.35  (28.78-50.97) | 32118  (24388-43374) | 26.34  (20.20-34.86) | 1119378  (847412-1520925) | 783.30  (597.30-1054.53) |
| Palau | 8  (6-11) | 36.19  (26.86-47.26) | 3  (3-5) | 17.15  (12.87-21.51) | 110  (80-145) | 464.41  (341.78-598.90) |
| Palestine | 845  (693-1019) | 29.35  (24.06-35.25) | 339  (280-405) | 13.46  (11.17-16.08) | 11225  (9233-13356) | 376.21  (310.70-447.25) |
| Panama | 925  (699-1183) | 22.10  (16.71-28.24) | 297  (228-376) | 7.07  (5.43-8.96) | 8637  (6503-11131) | 205.67  (155.03-264.77) |
| Papua New Guinea | 2078  (1508-2827) | 30.91  (22.87-41.47) | 1287  (944-1725) | 20.83  (15.61-27.67) | 49888  (36209-67731) | 704.24  (515.69-938.66) |
| Paraguay | 1348  (987-1737) | 22.79  (16.86-29.29) | 540  (409-686) | 9.50  (7.23-12.06) | 16306  (12042-21214) | 270.32  (201.01-350.28) |
| Peru | 4795  (3539-6383) | 14.43  (10.68-19.22) | 1872  (1420-2441) | 5.70  (4.32-7.43) | 55704  (40920-73973) | 166.46  (122.71-220.36) |
| Philippines | 19247  (14387-25103) | 21.25  (16.05-27.57) | 10255  (7741-13310) | 12.27  (9.42-15.75) | 343852  (257829-449078) | 369.34  (279.12-479.27) |
| Poland | 18756  (14673-23572) | 30.10  (23.58-38.26) | 7572  (6063-9372) | 11.09  (8.86-13.79) | 178755  (144376-222351) | 285.59  (229.76-356.66) |
| Portugal | 7505  (5793-9736) | 39.73  (30.34-52.01) | 2145  (1918-2358) | 9.32  (8.47-10.15) | 49474  (45014-54631) | 261.80  (238.87-290.02) |
| Puerto Rico | 2222  (1709-2826) | 38.74  (29.52-50.21) | 626  (491-787) | 9.54  (7.41-12.19) | 15750  (12189-20164) | 283.23  (215.60-364.92) |
| Qatar | 388  (287-509) | 25.46  (19.80-32.10) | 88  (66-114) | 8.99  (7.08-11.17) | 3399  (2551-4461) | 209.71  (162.48-262.55) |
| Republic of Korea | 20328  (16423-24533) | 23.79  (19.29-28.65) | 4090  (3603-4578) | 4.69  (4.12-5.24) | 122960  (108643-138700) | 143.56  (126.99-161.59) |
| Republic of Moldova | 1245  (1052-1469) | 22.24  (18.89-26.47) | 512  (437-602) | 8.96  (7.64-10.48) | 14408  (12300-17075) | 259.44  (220.80-307.80) |
| Romania | 8751  (7089-10635) | 27.35  (22.12-33.38) | 3661  (3007-4417) | 10.38  (8.49-12.61) | 92126  (74533-112708) | 289.09  (233.21-355.43) |
| Russian Federation | 68411  (55984-83181) | 31.26  (25.43-38.26) | 23778  (19601-28319) | 10.40  (8.55-12.41) | 643993  (535844-773926) | 293.62  (243.31-354.27) |
| Rwanda | 1118  (816-1500) | 16.78  (12.78-21.91) | 760  (572-998) | 12.77  (9.87-16.27) | 23999  (17398-32941) | 329.49  (245.77-436.32) |
| Saint Kitts and Nevis | 31  (23-40) | 44.28  (32.78-56.93) | 11  (8-13) | 16.74  (13.39-20.79) | 305  (218-400) | 423.33  (306.49-550.70) |
| Saint Lucia | 64  (53-77) | 29.19  (24.39-34.99) | 26  (21-30) | 11.98  (10.07-14.11) | 739  (612-884) | 335.82  (278.75-400.93) |
| Saint Vincent and the Grenadines | 45  (38-53) | 33.68  (28.73-39.72) | 20  (18-24) | 15.58  (13.47-17.96) | 593  (505-698) | 441.60  (376.78-519.61) |
| Samoa | 35  (23-57) | 21.96  (14.65-35.02) | 19  (13-30) | 12.47  (8.53-19.70) | 604  (395-960) | 369.00  (243.79-585.35) |
| San Marino | 24  (18-33) | 44.35  (32.71-61.16) | 7  (4-10) | 10.51  (6.82-15.76) | 155  (101-235) | 292.89  (189.39-452.95) |
| Sao Tome and Principe | 21  (13-31) | 16.46  (10.38-24.35) | 12  (8-18) | 10.83  (6.82-15.94) | 399  (251-595) | 300.82  (189.94-446.16) |
| Saudi Arabia | 5371  (3870-7305) | 17.16  (12.80-22.70) | 1428  (1049-1890) | 5.85  (4.47-7.60) | 56580  (40898-76008) | 177.80  (132.72-235.10) |
| Senegal | 1281  (894-1678) | 15.22  (10.90-19.90) | 875  (622-1146) | 11.21  (8.11-14.59) | 28053  (19361-37362) | 314.17  (218.85-413.50) |
| Serbia | 6253  (4901-8029) | 44.44  (34.59-57.03) | 2394  (1920-2978) | 16.01  (12.82-19.86) | 58677  (46385-74089) | 419.27  (330.52-531.43) |
| Seychelles | 33  (27-39) | 27.39  (22.54-32.51) | 15  (12-18) | 13.09  (10.90-15.45) | 457  (376-553) | 374.19  (309.62-448.46) |
| Sierra Leone | 502  (353-724) | 12.00  (8.60-17.25) | 345  (247-497) | 8.99  (6.56-12.92) | 11467  (7919-16837) | 254.36  (178.85-368.50) |
| Singapore | 2524  (1994-3142) | 30.97  (24.52-38.48) | 515  (465-560) | 6.49  (5.83-7.07) | 15636  (14178-17350) | 190.18  (172.16-211.06) |
| Slovakia | 2901  (2161-3825) | 33.38  (24.72-44.13) | 974  (733-1258) | 10.87  (8.18-14.04) | 24030  (17935-31778) | 278.40  (207.56-368.49) |
| Slovenia | 1233  (947-1638) | 33.69  (25.67-45.12) | 438  (339-570) | 10.00  (7.78-13.14) | 9338  (7225-12238) | 248.37  (191.67-328.10) |
| Solomon Islands | 281  (215-363) | 62.63  (48.30-80.33) | 153  (117-196) | 37.10  (29.18-46.81) | 6079  (4597-7916) | 1306.47  (1004.77-1673.33) |
| Somalia | 701  (428-1102) | 9.34  (5.68-14.58) | 571  (355-889) | 8.33  (5.16-12.90) | 19412  (12170-30091) | 224.52  (138.82-351.48) |
| South Africa | 8682  (7506-9966) | 18.70  (16.37-21.34) | 5309  (4671-6033) | 12.44  (11.05-14.02) | 147253  (128115-169348) | 303.18  (264.90-347.26) |
| South Sudan | 380  (252-539) | 8.63  (5.97-11.91) | 291  (192-416) | 7.27  (5.04-9.98) | 9474  (6002-14071) | 192.86  (126.27-277.78) |
| Spain | 29815  (23127-38294) | 36.96  (28.46-47.79) | 8075  (7100-8851) | 8.39  (7.60-9.06) | 186731  (169266-204238) | 233.69  (213.36-255.81) |
| Sri Lanka | 4168  (3060-5609) | 16.29  (12.03-21.92) | 1692  (1248-2246) | 6.86  (5.10-9.08) | 48585  (35693-65099) | 188.35  (138.15-251.93) |
| Sudan | 2902  (1865-4062) | 11.77  (7.97-16.19) | 1414  (970-1954) | 6.38  (4.60-8.60) | 51833  (33183-74982) | 204.71  (138.13-285.62) |
| Suriname | 131  (104-162) | 21.18  (16.86-25.84) | 63  (51-77) | 10.51  (8.49-12.65) | 1864  (1481-2316) | 297.15  (237.54-365.35) |
| Sweden | 7320  (5846-9046) | 42.17  (33.38-52.36) | 1947  (1724-2110) | 9.43  (8.57-10.18) | 42899  (39125-46568) | 250.25  (229.78-270.59) |
| Switzerland | 6160  (4734-7936) | 40.22  (30.66-52.09) | 1697  (1472-1859) | 9.64  (8.53-10.49) | 37774  (33790-41646) | 249.11  (224.80-275.01) |
| Syrian Arab Republic | 1882  (1311-2638) | 13.43  (9.39-18.65) | 715  (504-1010) | 5.42  (3.87-7.51) | 24137  (16868-34521) | 168.02  (119.20-237.24) |
| Taiwan  (Province of China) | 9330  (7069-12459) | 25.55  (19.26-34.00) | 2333  (1828-3015) | 6.09  (4.75-7.87) | 73595  (56993-96000) | 199.78  (154.67-261.88) |
| Tajikistan | 862  (666-1107) | 13.52  (10.59-17.19) | 445  (345-567) | 8.02  (6.38-10.09) | 15702  (12018-20223) | 232.41  (181.40-294.85) |
| Thailand | 17739  (12784-23841) | 17.57  (12.65-23.51) | 6834  (5022-8995) | 6.70  (4.90-8.79) | 210771  (152046-282030) | 207.90  (149.88-278.24) |
| Timor-Leste | 109  (73-150) | 12.44  (8.65-17.11) | 68  (48-94) | 8.18  (5.88-11.24) | 2174  (1411-3052) | 244.24  (162.62-342.90) |
| Togo | 596  (438-811) | 13.90  (10.64-18.45) | 392  (295-523) | 10.29  (7.99-13.52) | 13084  (9505-17798) | 278.35  (207.99-373.05) |
| Tokelau | 0  (0-1) | 31.79  (21.81-44.51) | 0  (0-0) | 16.86  (11.74-22.86) | 7  (5-9) | 499.28  (339.53-702.05) |
| Tonga | 24  (18-32) | 29.35  (21.66-38.54) | 14  (11-18) | 17.46  (13.19-22.63) | 416  (305-555) | 499.17  (366.88-662.35) |
| Trinidad and Tobago | 545  (401-732) | 29.86  (21.89-39.95) | 230  (174-299) | 12.65  (9.62-16.39) | 6457  (4763-8578) | 354.86  (261.07-472.49) |
| Tunisia | 3172  (2272-4293) | 23.90  (17.22-32.23) | 1061  (771-1416) | 8.32  (6.07-11.00) | 33687  (24084-45533) | 251.08  (179.72-337.40) |
| Turkey | 17380  (13655-21843) | 18.89  (14.88-23.76) | 6049  (4828-7484) | 6.82  (5.46-8.43) | 179410  (141036-225876) | 193.11  (152.37-242.19) |
| Turkmenistan | 716  (560-931) | 15.21  (11.93-19.58) | 301  (237-383) | 6.87  (5.43-8.65) | 10752  (8441-13933) | 224.26  (176.97-288.53) |
| Tuvalu | 3  (2-4) | 29.72  (20.61-42.44) | 2  (1-3) | 18.49  (13.03-25.87) | 57  (39-82) | 529.80  (367.30-761.58) |
| Uganda | 3145  (2342-4053) | 19.09  (14.51-23.98) | 2112  (1596-2696) | 14.19  (10.97-17.66) | 69746  (51636-91567) | 389.10  (292.67-502.76) |
| Ukraine | 17869  (13990-22466) | 26.00  (20.26-32.83) | 8561  (6796-10688) | 11.83  (9.37-14.83) | 242124  (192649-303818) | 355.16  (281.50-447.05) |
| United Arab Emirates | 1271  (906-1738) | 15.03  (11.50-19.41) | 455  (332-609) | 7.28  (5.61-9.32) | 18606  (13399-25270) | 204.37  (156.21-266.21) |
| United Kingdom | 53010  (42241-65883) | 49.21  (38.91-61.75) | 15256  (13900-16106) | 12.28  (11.37-12.88) | 340837  (319900-362996) | 319.94  (302.27-340.85) |
| United Republic of Tanzania | 3728  (2886-4627) | 13.83  (11.06-16.76) | 2602  (2073-3165) | 10.71  (8.70-12.73) | 79060  (60868-99360) | 269.38  (211.32-331.18) |
| United States of America | 254486  (210821-308184) | 50.22  (41.23-61.11) | 55021  (51008-57900) | 10.07  (9.45-10.55) | 1403392  (1314386-1499020) | 284.10  (267.11-303.32) |
| United States Virgin Islands | 70  (56-85) | 41.95  (33.17-52.07) | 28  (23-33) | 16.51  (13.42-19.91) | 694  (550-849) | 425.43  (332.30-527.88) |
| Uruguay | 1959  (1519-2470) | 40.27  (31.05-51.22) | 936  (849-1018) | 17.31  (15.83-18.70) | 21141  (19489-22931) | 446.23  (411.65-486.61) |
| Uzbekistan | 5480  (4449-6676) | 20.22  (16.65-24.19) | 2253  (1845-2704) | 9.74  (8.19-11.51) | 81133  (66329-98398) | 288.78  (238.28-345.84) |
| Vanuatu | 37  (24-55) | 18.60  (12.15-27.54) | 24  (16-35) | 13.21  (8.61-19.01) | 825  (523-1242) | 398.72  (259.27-592.68) |
| Venezuela  (Bolivarian Republic of) | 8475  (6289-11298) | 27.87  (20.78-37.03) | 2811  (2128-3671) | 9.47  (7.22-12.36) | 86571  (64614-116284) | 282.03  (211.58-377.79) |
| Viet Nam | 27821  (20840-36275) | 26.71  (20.23-34.41) | 11969  (9140-15527) | 12.41  (9.44-16.00) | 377467  (282930-491607) | 356.20  (268.47-461.97) |
| Yemen | 2032  (1446-2836) | 11.66  (8.55-16.02) | 1107  (810-1539) | 6.98  (5.21-9.50) | 40523  (28761-57471) | 222.87  (162.64-311.81) |
| Zambia | 1256  (915-1673) | 15.59  (11.74-20.56) | 812  (600-1074) | 11.58  (8.95-14.95) | 27629  (19665-37182) | 308.75  (226.84-410.42) |
| Zimbabwe | 1754  (1203-2432) | 21.64  (15.19-29.57) | 1161  (803-1614) | 15.84  (11.10-21.76) | 38520  (26126-54815) | 443.97  (304.25-622.08) |

ASIR, age-standardized incident rates; ASMR, age-standardized mortality rates; DALY, disability adjusted life-year; UI, uncertain interval.
