# Supplementary material for: The Global, Regional, and National Burden and Trends of Breast Cancer From 1990 to 2019: Results From the Global Burden of Disease Study 2019
Source: Front Oncol. 2021 May 21;11:689562. doi: 10.3389/fonc.2021.689562 (PMC8176863; doi:10.3389/fonc.2021.689562)
Supplement: Supplementary file 6 [file Table_2.docx]

**Supplementary Table 2. Breast cancer incident cases, age-standardized incidence rate, deaths, age-standardized mortality rate, DALYs, and age-standardized DALY rates in 2019.**

| **Characteristics** | **Relative change in incident cases (95% UI)** | **EAPC of ASIR (95% CI)** | **Relative change in deaths (95% UI)** | **EAPC of ASMR (95% CI)** | **Relative change in DALYs (95% UI)** | **EAPC of age-standardized DALY rates (95% CI)** |
| --- | --- | --- | --- | --- | --- | --- |
| Afghanistan | 196.12%  (106.34%-313.58%) | 0.85  (0.79-0.92) | 148.98%  (74.15%-242.27%) | 0.56  (0.52-0.59) | 169.72%  (88.68%-282.31%) | 0.26  (0.21-0.30) |
| Albania | 238.20%  (144.18%-365.68%) | 3.05  (2.75-3.36) | 128.40%  (68.21%-206.46%) | 1.07  (0.81-1.32) | 93.96%  (41.12%-164.14%) | 1.16  (0.88-1.44) |
| Algeria | 309.74%  (193.66%-467.24%) | 1.12  (1.02-1.20) | 171.35%  (96.02%-263.88%) | -0.27  (-0.38--0.16) | 167.27%  (91.20%-266.35%) | -0.21  (-0.29--0.13) |
| American Samoa | 223.81%  (128.94%-371.35%) | 2.02  (1.87-2.18) | 198.59%  (117.93%-330.46%) | 1.39  (1.29-1.49) | 169.62%  (89.74%-296.40%) | 1.46  (1.35-1.57) |
| Andorra | 207.02%  (92.88%-359.39%) | 0.85  (0.75-0.95) | 141.98%  (55.58%-250.47%) | -0.54  (-0.74--0.33) | 112.21%  (36.49%-212.43%) | -0.43  (-0.60--0.26) |
| Angola | 399.07%  (227.96%-666.71%) | 1.90  (1.82-1.98) | 319.36%  (172.53%-529.33%) | 1.43  (1.37-1.49) | 304.68%  (153.01%-534.55%) | 1.18  (1.11-1.25) |
| Antigua and Barbuda | 214.89%  (155.02%-284.75%) | 1.33  (1.19-1.47) | 134.59%  (94.77%-179.85%) | 0.55  (0.38-0.72) | 145.09%  (100.02%-196.96%) | 0.35  (0.19-0.52) |
| Argentina | 99.50%  (54.56%-152.35%) | 0.44  (0.23-0.64) | 54.88%  (43.46%-66.73%) | -0.51  (-0.66--0.35) | 39.72%  (29.47%-51.08%) | -0.67  (-0.80--0.54) |
| Armenia | 47.09%  (18.95%-80.68%) | -0.18  (-0.49-0.14) | 18.69%  (-1.63%-42.60%) | -1.18  (-1.50--0.86) | -0.47%  (-18.89%-20.45%) | -1.59  (-1.92--1.24) |
| Australia | 96.28%  (52.15%-152.81%) | -0.30  (-0.53--0.08) | 42.48%  (31.22%-53.90%) | -1.61  (-1.72--1.49) | 26.11%  (16.69%-37.23%) | -1.69  (-1.79--1.59) |
| Austria | 24.17%  (-0.03%-53.91%) | -0.69  (-0.80--0.57) | 3.94%  (-4.02%-12.07%) | -1.55  (-1.60--1.50) | -10.82%  (-17.39%--3.50%) | -1.85  (-1.91--1.79) |
| Azerbaijan | 141.50%  (87.67%-205.35%) | 0.54  (0.27-0.81) | 81.32%  (40.89%-127.92%) | -0.27  (-0.47--0.06) | 80.58%  (40.57%-127.81%) | -0.60  (-0.77--0.44) |
| Bahamas | 181.01%  (115.98%-263.27%) | 0.88  (0.71-1.05) | 140.92%  (92.16%-203.36%) | 0.22  (0.11-0.33) | 126.16%  (74.89%-191.36%) | 0.14  (0.04-0.24) |
| Bahrain | 639.82%  (457.55%-881.17%) | 0.88  (0.49-1.27) | 322.00%  (221.69%-448.49%) | -0.67  (-1.03--0.30) | 319.67%  (214.94%-452.69%) | -1.21  (-1.59--0.83) |
| Bangladesh | 253.02%  (152.57%-411.85%) | 0.79  (0.62-0.97) | 164.43%  (92.11%-274.52%) | -0.24  (-0.42--0.06) | 137.58%  (68.22%-247.81%) | -0.44  (-0.62--0.27) |
| Barbados | 149.28%  (99.19%-205.59%) | 1.45  (1.26-1.64) | 93.75%  (58.46%-132.35%) | 0.65  (0.48-0.81) | 91.43%  (53.34%-133.83%) | 0.49  (0.36-0.62) |
| Belarus | 31.53%  (-0.33%-71.92%) | -0.22  (-0.43--0.01) | -5.71%  (-27.08%-20.81%) | -1.61  (-1.88--1.32) | -15.45%  (-35.59%-11.23%) | -1.88  (-2.15--1.62) |
| Belgium | 27.51%  (-0.95%-64.18%) | -0.51  (-0.66--0.35) | -1.51%  (-9.35%-6.88%) | -1.81  (-1.92--1.71) | -14.80%  (-21.63%--7.16%) | -1.99  (-2.08--1.89) |
| Belize | 392.88%  (296.15%-506.56%) | 1.39  (1.06-1.73) | 267.74%  (204.12%-345.39%) | 0.58  (0.26-0.90) | 318.78%  (242.11%-414.38%) | 0.73  (0.42-1.05) |
| Benin | 260.03%  (162.60%-389.65%) | 1.13  (1.07-1.19) | 215.00%  (135.81%-320.56%) | 0.83  (0.78-0.90) | 221.13%  (129.59%-344.02%) | 0.60  (0.54-0.67) |
| Bermuda | 53.13%  (20.49%-98.80%) | -1.10  (-1.30--0.92) | 10.76%  (-10.16%-39.98%) | -2.70  (-2.91--2.49) | -9.51%  (-27.83%-16.74%) | -2.83  (-3.05--2.61) |
| Bhutan | 178.73%  (83.40%-339.04%) | 0.88  (0.68-1.09) | 101.28%  (35.73%-206.39%) | -0.32  (-0.50--0.14) | 76.34%  (14.43%-180.84%) | -0.66  (-0.86--0.46) |
| Bolivia  (Plurinational State of) | 299.84%  (192.75%-461.06%) | 1.24  (1.14-1.34) | 193.70%  (118.40%-301.32%) | 0.20  (0.13-0.27) | 165.71%  (91.61%-278.34%) | -0.07  (-0.15-0.01) |
| Bosnia and Herzegovina | 152.85%  (91.32%-228.23%) | 2.99  (2.63-3.35) | 106.18%  (59.69%-161.77%) | 1.76  (1.45-2.05) | 60.26%  (21.74%-104.97%) | 1.18  (0.96-1.39) |
| Botswana | 396.05%  (184.87%-683.12%) | 2.35  (2.15-2.55) | 267.83%  (116.49%-472.49%) | 1.50  (1.25-1.75) | 275.47%  (108.06%-510.93%) | 1.45  (1.22-1.68) |
| Brazil | 210.94%  (190.56%-233.11%) | 0.75  (0.52-0.99) | 126.52%  (111.83%-141.84%) | -0.52  (-0.69--0.36) | 107.29%  (94.80%-121.28%) | -0.50  (-0.67--0.33) |
| Brunei Darussalam | 350.22%  (229.05%-500.96%) | 2.36  (2.11-2.60) | 236.32%  (154.57%-332.22%) | 1.31  (1.06-1.56) | 208.54%  (128.93%-305.88%) | 1.10  (0.79-1.41) |
| Bulgaria | 51.42%  (17.34%-94.95%) | 2.34  (1.91-2.77) | 35.18%  (6.22%-69.02%) | 1.70  (1.26-2.14) | 11.77%  (-12.94%-42.81%) | 1.27  (0.87-1.67) |
| Burkina Faso | 198.98%  (120.52%-300.24%) | 0.88  (0.73-1.05) | 160.65%  (93.96%-245.50%) | 0.51  (0.32-0.69) | 167.36%  (91.90%-266.95%) | 0.42  (0.21-0.62) |
| Burundi | 86.77%  (22.59%-194.09%) | -0.69  (-0.82--0.56) | 63.90%  (8.48%-151.00%) | -1.00  (-1.15--0.85) | 68.60%  (7.24%-173.20%) | -1.25  (-1.43--1.08) |
| Cabo Verde | 189.05%  (111.74%-279.35%) | 1.16  (0.83-1.49) | 120.58%  (63.02%-187.07%) | 0.33  (0.06-0.60) | 102.70%  (49.41%-168.06%) | -0.33  (-0.59--0.07) |
| Cambodia | 297.63%  (171.45%-457.49%) | 1.58  (1.53-1.64) | 208.95%  (110.90%-327.26%) | 0.71  (0.65-0.79) | 178.76%  (87.23%-298.69%) | 0.37  (0.29-0.45) |
| Cameroon | 309.11%  (173.88%-521.88%) | 1.33  (1.25-1.41) | 245.85%  (134.61%-428.09%) | 0.90  (0.83-0.98) | 247.84%  (126.65%-433.75%) | 0.71  (0.63-0.80) |
| Canada | 72.00%  (33.88%-119.33%) | -0.63  (-0.72--0.53) | 40.79%  (29.96%-52.28%) | -1.67  (-1.75--1.58) | 23.81%  (13.56%-34.98%) | -1.75  (-1.86--1.66) |
| Central African Republic | 105.44%  (34.28%-232.61%) | 0.28  (0.23-0.33) | 99.70%  (31.51%-220.83%) | 0.21  (0.16-0.27) | 100.27%  (30.46%-225.22%) | 0.07  (0.01-0.13) |
| Chad | 171.72%  (103.19%-270.32%) | 0.74  (0.72-0.77) | 145.10%  (86.64%-232.42%) | 0.53  (0.49-0.57) | 159.43%  (90.14%-264.54%) | 0.45  (0.41-0.49) |
| Chile | 183.81%  (120.04%-266.41%) | 1.08  (0.98-1.17) | 90.55%  (73.57%-108.50%) | -0.70  (-0.75--0.64) | 66.08%  (49.72%-83.47%) | -0.79  (-0.88--0.69) |
| China | 360.02%  (238.44%-514.60%) | 2.84  (2.74-2.95) | 130.38%  (76.42%-201.92%) | 0.06  (0.00-0.12) | 106.08%  (58.39%-172.63%) | -0.13  (-0.19--0.06) |
| Colombia | 270.27%  (185.83%-381.46%) | 1.21  (1.10-1.32) | 135.19%  (84.43%-202.30%) | -0.64  (-0.77--0.51) | 115.21%  (66.30%-179.07%) | -0.46  (-0.60--0.31) |
| Comoros | 243.62%  (120.54%-599.17%) | 1.37  (1.21-1.52) | 198.02%  (98.60%-466.41%) | 0.85  (0.71-1.00) | 188.29%  (79.20%-558.75%) | 0.69  (0.48-0.90) |
| Congo | 229.04%  (114.48%-395.05%) | 0.62  (0.48-0.76) | 179.06%  (84.04%-317.43%) | 0.14  (-0.01-0.28) | 184.57%  (83.88%-330.82%) | -0.02  (-0.18-0.14) |
| Cook Islands | 104.84%  (39.11%-192.12%) | 0.92  (0.69-1.15) | 81.65%  (29.52%-155.90%) | 0.10  (-0.12-0.32) | 48.33%  (0.09%-112.53%) | -0.08  (-0.35-0.18) |
| Costa Rica | 296.23%  (201.25%-409.06%) | 1.46  (1.32-1.61) | 212.77%  (144.31%-299.23%) | 0.36  (0.22-0.49) | 173.80%  (108.19%-252.80%) | 0.15  (0.00-0.29) |
| Croatia | 28.22%  (-2.26%-64.86%) | 0.40  (0.19-0.61) | 11.87%  (-12.09%-38.88%) | -0.51  (-0.73--0.30) | -10.97%  (-31.28%-14.06%) | -0.98  (-1.17--0.77) |
| Cuba | 113.43%  (70.55%-164.76%) | 0.75  (0.63-0.87) | 74.21%  (41.09%-111.42%) | -0.16  (-0.29--0.03) | 50.74%  (20.59%-87.03%) | -0.45  (-0.57--0.33) |
| Cyprus | 267.14%  (189.32%-366.73%) | 1.97  (1.65-2.29) | 108.26%  (69.42%-152.84%) | -0.32  (-0.42--0.21) | 94.45%  (57.82%-139.00%) | -0.54  (-0.63--0.44) |
| Czechia | 29.99%  (4.13%-61.52%) | -0.48  (-0.78--0.18) | -0.35%  (-18.12%-20.59%) | -1.61  (-1.82--1.39) | -14.31%  (-29.81%-4.28%) | -1.88  (-2.06--1.70) |
| Côte d'Ivoire | 257.58%  (141.99%-426.71%) | 1.25  (1.14-1.34) | 227.93%  (127.30%-369.91%) | 0.96  (0.85-1.08) | 215.54%  (109.80%-370.75%) | 0.82  (0.72-0.94) |
| Democratic People's Republic of Korea | 115.89%  (37.39%-240.26%) | 0.76  (0.66-0.88) | 107.07%  (36.23%-214.02%) | 0.41  (0.30-0.53) | 90.90%  (20.33%-198.56%) | 0.36  (0.26-0.45) |
| Democratic Republic of the Congo | 212.10%  (114.61%-341.94%) | 0.97  (0.80-1.16) | 187.65%  (97.73%-306.36%) | 0.81  (0.66-0.97) | 175.95%  (88.38%-290.09%) | 0.51  (0.35-0.67) |
| Denmark | 18.06%  (-9.08%-50.42%) | -0.42  (-0.66--0.18) | -16.09%  (-23.83%--8.03%) | -2.13  (-2.23--2.01) | -27.76%  (-34.59%--20.67%) | -2.52  (-2.62--2.42) |
| Djibouti | 530.88%  (297.08%-906.23%) | 1.52  (1.40-1.64) | 439.86%  (253.05%-756.11%) | 1.02  (0.96-1.08) | 418.77%  (217.00%-737.88%) | 0.88  (0.82-0.95) |
| Dominica | 37.34%  (7.25%-78.13%) | 0.30  (0.22-0.38) | 29.09%  (3.24%-65.11%) | 0.13  (0.04-0.22) | 27.97%  (-0.97%-66.66%) | -0.10  (-0.20-0.00) |
| Dominican Republic | 366.29%  (212.64%-560.92%) | 2.85  (2.56-3.14) | 255.90%  (149.32%-388.71%) | 1.99  (1.73-2.24) | 225.67%  (118.00%-362.13%) | 1.82  (1.57-2.07) |
| Ecuador | 439.53%  (319.32%-610.33%) | 2.69  (2.46-2.91) | 250.00%  (176.55%-354.70%) | 1.19  (0.96-1.41) | 224.54%  (153.50%-327.36%) | 1.04  (0.81-1.26) |
| Egypt | 322.20%  (187.36%-492.56%) | 2.17  (1.64-2.71) | 194.19%  (96.20%-314.07%) | 1.09  (0.57-1.61) | 185.72%  (92.81%-303.49%) | 1.01  (0.48-1.53) |
| El Salvador | 332.91%  (214.37%-500.63%) | 3.01  (2.79-3.24) | 183.97%  (110.24%-281.43%) | 1.35  (1.23-1.46) | 153.66%  (81.75%-250.12%) | 1.26  (1.16-1.36) |
| Equatorial Guinea | 510.18%  (215.17%-1081.42%) | 3.53  (3.35-3.71) | 344.35%  (134.49%-762.97%) | 2.52  (2.41-2.64) | 318.96%  (114.70%-711.50%) | 1.98  (1.86-2.10) |
| Eritrea | 344.78%  (187.53%-618.33%) | 1.80  (1.66-1.93) | 289.64%  (154.71%-517.33%) | 1.43  (1.28-1.58) | 270.47%  (137.39%-494.81%) | 1.22  (1.10-1.33) |
| Estonia | 36.41%  (3.41%-75.80%) | 0.66  (0.48-0.84) | -1.73%  (-23.17%-23.39%) | -1.14  (-1.32--0.97) | -19.52%  (-38.08%-3.63%) | -1.57  (-1.79--1.34) |
| Eswatini | 202.42%  (74.01%-409.72%) | 1.89  (1.48-2.29) | 172.36%  (60.33%-352.80%) | 1.63  (1.15-2.12) | 162.47%  (47.67%-350.49%) | 1.53  (1.02-2.05) |
| Ethiopia | 115.29%  (46.51%-226.77%) | -0.10  (-0.35-0.16) | 83.04%  (30.75%-164.95%) | -0.55  (-0.76--0.34) | 66.51%  (14.02%-158.58%) | -0.99  (-1.20--0.77) |
| Fiji | 142.85%  (62.85%-262.28%) | 1.40  (1.20-1.59) | 130.76%  (55.32%-244.87%) | 1.15  (0.97-1.31) | 103.80%  (36.41%-207.75%) | 0.90  (0.69-1.11) |
| Finland | 73.75%  (35.00%-124.22%) | 0.52  (0.27-0.78) | 23.20%  (11.25%-35.21%) | -1.30  (-1.38--1.22) | 4.12%  (-5.86%-14.45%) | -1.41  (-1.51--1.31) |
| France | 67.01%  (28.10%-114.20%) | 0.33  (0.01-0.65) | 21.32%  (9.12%-32.00%) | -1.36  (-1.53--1.19) | 6.44%  (-2.58%-15.80%) | -1.37  (-1.57--1.17) |
| Gabon | 177.93%  (78.78%-330.90%) | 0.98  (0.85-1.12) | 129.04%  (50.06%-251.34%) | 0.49  (0.34-0.65) | 131.40%  (48.42%-254.63%) | 0.28  (0.09-0.47) |
| Gambia | 443.25%  (221.47%-796.35%) | 2.39  (2.16-2.61) | 391.66%  (199.80%-695.29%) | 2.02  (1.81-2.22) | 373.73%  (179.51%-671.58%) | 2.02  (1.78-2.25) |
| Georgia | -16.91%  (-33.54%-2.19%) | 0.09  (-0.21-0.40) | -16.73%  (-32.85%-0.04%) | 0.19  (-0.17-0.55) | -28.12%  (-42.27%--12.54%) | -0.16  (-0.48-0.16) |
| Germany | 50.39%  (16.13%-92.77%) | 0.24  (0.07-0.41) | 12.50%  (3.03%-21.30%) | -1.21  (-1.30--1.13) | -3.83%  (-11.35%-4.25%) | -1.39  (-1.47--1.31) |
| Ghana | 283.83%  (164.20%-447.71%) | 1.17  (1.02-1.32) | 226.19%  (127.23%-360.53%) | 0.75  (0.63-0.89) | 212.11%  (114.64%-350.70%) | 0.58  (0.44-0.72) |
| Greece | 66.25%  (29.23%-114.76%) | 0.46  (0.27-0.64) | 56.17%  (41.58%-70.43%) | -0.51  (-0.68--0.33) | 19.11%  (8.63%-30.31%) | -0.69  (-0.76--0.63) |
| Greenland | 2.12%  (-23.94%-41.05%) | -2.33  (-2.60--2.08) | -28.29%  (-45.26%--3.94%) | -3.64  (-4.00--3.27) | -26.44%  (-44.90%-2.28%) | -3.16  (-3.40--2.92) |
| Grenada | 130.11%  (91.72%-171.15%) | 1.09  (0.81-1.36) | 68.90%  (44.58%-95.47%) | 0.59  (0.26-0.93) | 81.49%  (51.07%-114.33%) | 0.26  (-0.09-0.60) |
| Guam | 180.55%  (117.77%-256.66%) | 0.74  (0.50-0.99) | 173.65%  (114.39%-244.26%) | -0.03  (-0.35-0.29) | 143.06%  (89.24%-208.30%) | 0.55  (0.37-0.73) |
| Guatemala | 427.71%  (292.92%-623.98%) | 2.39  (2.21-2.57) | 282.51%  (192.02%-405.46%) | 1.15  (1.02-1.26) | 238.43%  (155.08%-360.94%) | 0.88  (0.74-1.02) |
| Guinea | 134.48%  (61.21%-237.07%) | 1.47  (1.40-1.54) | 110.91%  (47.00%-198.80%) | 1.21  (1.14-1.28) | 114.96%  (47.43%-208.65%) | 0.98  (0.92-1.06) |
| Guinea-Bissau | 174.38%  (85.99%-305.52%) | 1.05  (1.02-1.08) | 146.05%  (68.38%-260.70%) | 0.77  (0.73-0.80) | 145.51%  (65.93%-264.86%) | 0.68  (0.64-0.72) |
| Guyana | 113.17%  (52.75%-191.78%) | 0.97  (0.77-1.19) | 76.74%  (29.67%-136.28%) | 0.45  (0.28-0.63) | 75.55%  (25.60%-140.63%) | 0.58  (0.37-0.80) |
| Haiti | 184.24%  (94.59%-320.49%) | 0.99  (0.88-1.10) | 143.13%  (66.29%-261.44%) | 0.47  (0.37-0.57) | 143.87%  (64.40%-254.45%) | 0.48  (0.37-0.58) |
| Honduras | 434.03%  (252.80%-743.84%) | 2.31  (2.12-2.48) | 312.84%  (184.61%-530.97%) | 1.45  (1.25-1.66) | 267.46%  (141.84%-473.76%) | 1.08  (0.94-1.22) |
| Hungary | 28.68%  (3.67%-59.28%) | -0.27  (-0.58-0.04) | 1.22%  (-16.30%-21.43%) | -1.34  (-1.55--1.13) | -12.78%  (-28.85%-6.27%) | -1.55  (-1.76--1.34) |
| Iceland | 37.74%  (13.73%-64.25%) | -1.36  (-1.60--1.13) | 8.96%  (-5.36%-23.79%) | -2.39  (-2.54--2.25) | -0.59%  (-13.45%-13.19%) | -2.46  (-2.60--2.32) |
| India | 292.59%  (193.50%-408.84%) | 1.73  (1.54-1.92) | 210.17%  (132.71%-305.98%) | 0.75  (0.58-0.92) | 187.20%  (115.48%-276.03%) | 0.82  (0.64-1.00) |
| Indonesia | 198.91%  (117.33%-309.61%) | 1.29  (1.17-1.40) | 139.77%  (76.25%-222.71%) | 0.53  (0.41-0.66) | 126.18%  (64.77%-208.34%) | 0.40  (0.27-0.53) |
| Iran  (Islamic Republic of) | 417.63%  (292.69%-542.44%) | 1.98  (1.80-2.15) | 231.46%  (151.36%-314.34%) | 0.58  (0.41-0.75) | 222.01%  (150.06%-290.74%) | 0.63  (0.48-0.78) |
| Iraq | 484.98%  (268.42%-785.16%) | 2.08  (1.95-2.22) | 290.70%  (150.13%-490.20%) | 0.70  (0.62-0.77) | 297.69%  (153.82%-505.93%) | 0.61  (0.54-0.68) |
| Ireland | 85.00%  (40.66%-142.69%) | 0.14  (-0.02-0.29) | 17.88%  (7.59%-30.34%) | -1.72  (-1.77--1.68) | 9.31%  (-1.52%-22.22%) | -1.91  (-1.97--1.86) |
| Israel | 135.78%  (77.74%-202.76%) | -0.44  (-0.71--0.17) | 70.72%  (56.51%-85.45%) | -1.95  (-2.20--1.70) | 46.54%  (34.07%-58.49%) | -2.11  (-2.31--1.91) |
| Italy | 41.39%  (10.86%-81.24%) | -0.23  (-0.42--0.04) | 17.49%  (7.09%-24.67%) | -1.42  (-1.49--1.36) | -3.58%  (-9.34%-1.77%) | -1.60  (-1.66--1.53) |
| Jamaica | 210.11%  (139.22%-298.61%) | 2.02  (1.72-2.31) | 148.22%  (96.19%-208.77%) | 1.12  (0.82-1.42) | 165.00%  (104.86%-240.75%) | 1.40  (1.09-1.73) |
| Japan | 135.93%  (87.29%-193.22%) | 1.96  (1.72-2.20) | 101.77%  (77.85%-116.74%) | 0.55  (0.41-0.70) | 52.29%  (41.36%-61.91%) | 0.42  (0.24-0.60) |
| Jordan | 578.95%  (391.92%-845.71%) | 1.18  (0.91-1.45) | 319.48%  (206.88%-485.93%) | -0.50  (-0.82--0.18) | 300.79%  (191.66%-457.02%) | -0.79  (-1.17--0.41) |
| Kazakhstan | 58.13%  (30.51%-88.18%) | 1.06  (0.86-1.25) | 17.52%  (-1.20%-37.14%) | 0.01  (-0.32-0.34) | 12.51%  (-5.83%-31.42%) | -0.37  (-0.70--0.04) |
| Kenya | 350.24%  (218.39%-576.97%) | 1.56  (1.50-1.62) | 311.52%  (201.35%-497.35%) | 1.62  (1.56-1.69) | 324.18%  (206.09%-538.47%) | 1.53  (1.46-1.60) |
| Kiribati | 131.67%  (52.96%-243.85%) | 0.72  (0.62-0.81) | 113.92%  (44.43%-219.74%) | 0.56  (0.48-0.64) | 106.99%  (37.80%-206.64%) | 0.31  (0.25-0.38) |
| Kuwait | 392.51%  (284.79%-541.84%) | 0.99  (0.65-1.33) | 230.23%  (164.02%-330.85%) | -0.32  (-0.72-0.08) | 221.75%  (155.21%-320.32%) | -0.48  (-0.89--0.07) |
| Kyrgyzstan | 17.09%  (-1.54%-38.50%) | -1.55  (-1.92--1.18) | -9.06%  (-22.50%-5.34%) | -2.18  (-2.46--1.89) | -9.54%  (-23.41%-5.44%) | -2.55  (-2.85--2.24) |
| Lao People's Democratic Republic | 165.46%  (77.97%-285.94%) | 0.50  (0.41-0.58) | 111.16%  (45.14%-201.72%) | -0.19  (-0.25--0.13) | 106.26%  (37.57%-202.19%) | -0.39  (-0.45--0.33) |
| Latvia | 13.86%  (-15.79%-47.62%) | 0.67  (0.41-0.94) | -6.10%  (-27.37%-20.82%) | -0.48  (-0.73--0.23) | -23.94%  (-42.63%--1.25%) | -0.95  (-1.21--0.68) |
| Lebanon | 470.28%  (297.54%-699.36%) | 3.95  (3.70-4.19) | 221.00%  (125.58%-350.31%) | 1.64  (1.38-1.90) | 189.21%  (101.85%-306.59%) | 1.46  (1.25-1.67) |
| Lesotho | 207.32%  (67.07%-413.39%) | 3.95  (3.52-4.39) | 179.73%  (58.19%-358.64%) | 3.70  (3.25-4.14) | 192.58%  (57.31%-402.25%) | 3.87  (3.37-4.37) |
| Liberia | 213.77%  (101.25%-409.42%) | 1.54  (1.35-1.74) | 159.77%  (70.10%-320.96%) | 1.08  (0.89-1.26) | 178.81%  (78.54%-353.20%) | 0.85  (0.66-1.05) |
| Libya | 532.26%  (287.13%-890.44%) | 2.74  (2.46-3.02) | 347.44%  (182.92%-587.77%) | 1.51  (1.33-1.69) | 367.29%  (190.49%-636.22%) | 1.53  (1.33-1.73) |
| Lithuania | 14.12%  (-9.70%-42.75%) | 0.27  (0.06-0.48) | 7.56%  (-13.24%-30.28%) | -0.41  (-0.63--0.19) | -13.32%  (-30.69%-7.43%) | -0.80  (-1.01--0.57) |
| Luxembourg | 49.85%  (19.32%-87.53%) | -0.52  (-0.83--0.22) | 6.02%  (-8.71%-23.60%) | -2.02  (-2.13--1.90) | -2.71%  (-16.45%-13.03%) | -2.10  (-2.20--1.99) |
| Madagascar | 186.84%  (98.53%-300.89%) | 0.67  (0.59-0.76) | 156.40%  (80.62%-252.80%) | 0.45  (0.37-0.53) | 161.69%  (79.58%-262.35%) | 0.28  (0.18-0.37) |
| Malawi | 152.23%  (74.79%-258.59%) | 1.32  (1.21-1.43) | 133.95%  (66.86%-230.42%) | 1.16  (1.05-1.26) | 115.11%  (46.33%-209.59%) | 0.61  (0.52-0.70) |
| Malaysia | 351.28%  (231.23%-497.22%) | 1.83  (1.71-1.96) | 209.69%  (130.57%-303.12%) | 0.36  (0.24-0.49) | 186.85%  (113.74%-277.56%) | 0.31  (0.14-0.49) |
| Maldives | 308.74%  (156.23%-670.03%) | 0.38  (0.19-0.58) | 133.39%  (45.21%-318.72%) | -1.46  (-1.69--1.23) | 101.99%  (23.12%-277.83%) | -2.18  (-2.39--1.96) |
| Mali | 165.91%  (89.09%-268.40%) | 0.49  (0.36-0.63) | 131.55%  (67.09%-214.08%) | 0.09  (-0.03-0.22) | 126.74%  (60.75%-213.45%) | -0.10  (-0.24-0.05) |
| Malta | 64.46%  (34.02%-103.33%) | -0.41  (-0.53--0.28) | 20.16%  (2.73%-40.50%) | -2.08  (-2.17--2.00) | 3.05%  (-11.43%-20.55%) | -1.99  (-2.06--1.93) |
| Marshall Islands | 291.36%  (152.18%-492.25%) | 1.82  (1.60-2.04) | 240.91%  (124.43%-418.13%) | 1.33  (1.09-1.58) | 249.46%  (123.97%-427.37%) | 1.48  (1.23-1.74) |
| Mauritania | 154.73%  (72.55%-265.32%) | 0.62  (0.50-0.74) | 104.79%  (41.84%-184.89%) | -0.05  (-0.14-0.03) | 101.70%  (34.85%-193.70%) | -0.12  (-0.22--0.02) |
| Mauritius | 391.87%  (274.70%-528.00%) | 3.04  (2.78-3.29) | 305.37%  (221.35%-409.89%) | 2.16  (1.96-2.37) | 263.45%  (183.81%-358.65%) | 2.17  (1.95-2.39) |
| Mexico | 316.80%  (237.66%-411.31%) | 1.57  (1.40-1.75) | 185.40%  (135.39%-246.19%) | 0.27  (0.19-0.34) | 167.34%  (121.44%-225.23%) | 0.27  (0.18-0.35) |
| Micronesia  (Federated States of) | 182.96%  (51.26%-438.22%) | 2.15  (1.99-2.31) | 132.61%  (33.45%-338.19%) | 1.55  (1.42-1.69) | 125.53%  (15.99%-327.27%) | 1.37  (1.22-1.51) |
| Monaco | 83.52%  (29.08%-158.32%) | 1.50  (1.27-1.73) | 41.95%  (3.64%-92.00%) | 0.33  (0.17-0.48) | 38.33%  (-1.54%-93.06%) | 0.35  (0.21-0.49) |
| Mongolia | 279.53%  (154.84%-445.90%) | 1.27  (0.96-1.58) | 179.50%  (95.09%-295.73%) | 0.44  (0.06-0.82) | 183.73%  (89.53%-312.53%) | -0.02  (-0.39-0.35) |
| Montenegro | 96.10%  (46.32%-154.33%) | 1.38  (1.24-1.53) | 64.03%  (23.16%-108.22%) | 0.43  (0.24-0.63) | 51.22%  (13.11%-95.03%) | 0.34  (0.10-0.59) |
| Morocco | 313.53%  (180.03%-512.48%) | 2.07  (2.03-2.12) | 195.95%  (101.37%-325.07%) | 0.93  (0.85-1.01) | 185.96%  (92.63%-321.78%) | 0.86  (0.77-0.96) |
| Mozambique | 219.43%  (115.35%-355.56%) | 2.11  (1.80-2.43) | 178.47%  (90.50%-293.90%) | 1.77  (1.47-2.06) | 172.54%  (79.35%-295.75%) | 1.52  (1.17-1.89) |
| Myanmar | 10.83%  (-25.37%-65.14%) | -2.03  (-2.32--1.74) | -7.76%  (-37.00%-34.17%) | -2.61  (-2.90--2.31) | -21.26%  (-47.95%-21.92%) | -3.18  (-3.51--2.85) |
| Namibia | 374.65%  (203.26%-644.04%) | 3.29  (3.17-3.42) | 267.34%  (145.11%-457.82%) | 2.41  (2.25-2.57) | 261.05%  (129.21%-475.23%) | 2.31  (2.14-2.48) |
| Nauru | 102.80%  (28.62%-277.27%) | 1.94  (1.88-2.00) | 65.87%  (6.84%-209.94%) | 1.42  (1.22-1.62) | 68.87%  (6.38%-214.15%) | 1.50  (1.30-1.70) |
| Nepal | 294.48%  (162.69%-541.40%) | 2.17  (1.99-2.35) | 208.77%  (105.44%-409.21%) | 1.31  (1.14-1.49) | 186.16%  (87.83%-373.68%) | 1.14  (0.95-1.33) |
| Netherlands | 56.26%  (20.04%-100.21%) | -0.25  (-0.38--0.12) | 20.26%  (10.29%-30.10%) | -1.54  (-1.68--1.40) | 5.40%  (-3.14%-14.59%) | -1.73  (-1.87--1.61) |
| New Zealand | 65.18%  (26.42%-112.71%) | -0.18  (-0.29--0.07) | 17.04%  (7.51%-27.36%) | -1.90  (-2.01--1.79) | 10.01%  (1.03%-19.96%) | -1.76  (-1.87--1.65) |
| Nicaragua | 546.88%  (398.32%-744.93%) | 3.24  (3.03-3.44) | 338.65%  (245.22%-462.05%) | 1.70  (1.37-2.03) | 273.56%  (187.84%-391.74%) | 1.24  (1.08-1.40) |
| Niger | 250.53%  (146.29%-382.87%) | 0.74  (0.64-0.84) | 213.96%  (122.97%-328.82%) | 0.35  (0.24-0.46) | 199.81%  (105.21%-317.00%) | 0.20  (0.07-0.33) |
| Nigeria | 295.28%  (135.64%-509.88%) | 2.34  (2.12-2.56) | 223.14%  (95.64%-392.82%) | 1.65  (1.46-1.84) | 243.60%  (103.87%-440.71%) | 1.79  (1.59-1.98) |
| Niue | 53.38%  (-2.94%-136.60%) | 1.56  (1.43-1.69) | 12.28%  (-25.57%-65.65%) | 0.48  (0.41-0.57) | 14.86%  (-27.18%-75.53%) | 0.40  (0.32-0.49) |
| North Macedonia | 134.59%  (68.00%-213.01%) | 1.45  (1.21-1.68) | 79.31%  (34.61%-133.15%) | 0.51  (0.32-0.71) | 49.78%  (10.35%-98.28%) | -0.16  (-0.32-0.01) |
| Northern Mariana Islands | 155.05%  (78.07%-282.67%) | 0.85  (0.68-1.02) | 155.92%  (83.07%-267.89%) | 0.40  (0.27-0.53) | 102.14%  (40.24%-197.93%) | 0.33  (0.21-0.44) |
| Norway | 40.94%  (13.00%-77.18%) | -0.07  (-0.46-0.33) | -8.86%  (-14.97%--1.73%) | -1.92  (-2.07--1.77) | -13.84%  (-19.43%--6.75%) | -2.08  (-2.26--1.89) |
| Oman | 497.06%  (297.18%-783.57%) | 2.58  (2.05-3.12) | 231.58%  (121.72%-387.88%) | 1.12  (0.79-1.44) | 240.24%  (127.62%-399.97%) | 0.77  (0.31-1.25) |
| Pakistan | 301.20%  (168.44%-562.12%) | 2.08  (1.92-2.25) | 227.49%  (112.57%-449.08%) | 1.53  (1.33-1.74) | 243.56%  (119.69%-471.63%) | 1.40  (1.18-1.63) |
| Palau | 171.80%  (77.11%-298.61%) | 0.96  (0.88-1.06) | 122.94%  (47.79%-222.85%) | 0.34  (0.23-0.45) | 113.66%  (38.09%-214.28%) | 0.21  (0.07-0.35) |
| Palestine | 376.15%  (199.73%-618.43%) | 1.68  (1.35-2.03) | 249.30%  (120.33%-427.01%) | 0.95  (0.71-1.20) | 247.87%  (121.85%-420.06%) | 0.61  (0.35-0.86) |
| Panama | 287.20%  (188.36%-403.59%) | 1.43  (1.23-1.63) | 181.31%  (113.81%-259.30%) | 0.41  (0.21-0.61) | 155.92%  (89.65%-230.56%) | 0.34  (0.12-0.56) |
| Papua New Guinea | 255.42%  (148.38%-402.50%) | 0.84  (0.80-0.89) | 217.48%  (125.61%-347.82%) | 0.56  (0.53-0.60) | 221.25%  (125.17%-354.78%) | 0.55  (0.51-0.58) |
| Paraguay | 370.25%  (231.93%-540.51%) | 2.15  (1.98-2.34) | 242.10%  (147.30%-356.62%) | 1.17  (0.96-1.37) | 223.38%  (127.72%-338.63%) | 1.01  (0.80-1.21) |
| Peru | 243.07%  (140.63%-380.14%) | 0.84  (0.65-1.05) | 119.83%  (58.96%-203.74%) | -0.92  (-1.14--0.70) | 98.02%  (39.26%-179.84%) | -1.04  (-1.26--0.82) |
| Philippines | 199.17%  (117.95%-312.91%) | 0.20  (-0.09-0.49) | 155.96%  (90.46%-246.60%) | -0.46  (-0.74--0.17) | 155.50%  (89.59%-247.60%) | -0.18  (-0.43-0.08) |
| Poland | 94.03%  (51.38%-143.84%) | 1.04  (0.86-1.22) | 43.00%  (15.84%-76.24%) | -0.58  (-0.70--0.46) | 19.69%  (-3.31%-48.28%) | -0.83  (-0.94--0.71) |
| Portugal | 69.77%  (30.48%-121.71%) | 0.19  (-0.01-0.40) | 17.97%  (6.81%-30.47%) | -1.73  (-1.82--1.65) | -2.71%  (-11.70%-7.15%) | -1.78  (-1.87--1.69) |
| Puerto Rico | 116.08%  (64.14%-183.44%) | 1.07  (0.93-1.21) | 69.37%  (33.06%-115.43%) | -0.41  (-0.53--0.30) | 39.78%  (7.21%-81.79%) | -0.55  (-0.67--0.43) |
| Qatar | 1175.49%  (765.73%-1716.91%) | 1.94  (1.53-2.33) | 521.73%  (332.33%-770.31%) | -0.37  (-0.72--0.02) | 533.50%  (333.42%-803.92%) | -0.57  (-0.87--0.27) |
| Republic of Korea | 487.52%  (369.57%-614.57%) | 3.48  (3.12-3.85) | 186.30%  (146.78%-224.67%) | 0.46  (0.31-0.60) | 138.40%  (103.80%-172.62%) | 0.39  (0.26-0.52) |
| Republic of Moldova | 15.88%  (-2.55%-37.74%) | 0.29  (0.02-0.56) | -3.16%  (-17.22%-13.28%) | -0.48  (-0.78--0.19) | -17.58%  (-29.97%--3.40%) | -0.97  (-1.24--0.69) |
| Romania | 74.72%  (41.12%-113.83%) | 1.28  (1.13-1.43) | 39.81%  (14.18%-68.68%) | 0.16  (0.03-0.29) | 13.70%  (-7.16%-39.07%) | -0.23  (-0.36--0.10) |
| Russian Federation | 82.51%  (49.66%-121.98%) | 1.28  (1.04-1.53) | 42.45%  (18.26%-68.50%) | -0.02  (-0.41-0.36) | 26.76%  (5.34%-51.01%) | -0.32  (-0.70-0.07) |
| Rwanda | 125.77%  (45.03%-251.13%) | -0.10  (-0.36-0.15) | 87.52%  (23.31%-184.29%) | -0.65  (-0.88--0.42) | 76.48%  (8.67%-189.27%) | -1.16  (-1.44--0.88) |
| Saint Kitts and Nevis | 70.39%  (21.43%-128.87%) | -0.55  (-0.68--0.42) | 21.65%  (-7.53%-57.86%) | -1.08  (-1.26--0.91) | 27.22%  (-10.63%-70.72%) | -1.72  (-1.91--1.52) |
| Saint Lucia | 147.36%  (95.32%-209.55%) | -0.28  (-0.55--0.01) | 89.95%  (55.66%-129.21%) | -1.31  (-1.64--0.98) | 84.62%  (47.98%-128.55%) | -1.17  (-1.50--0.84) |
| Saint Vincent and the Grenadines | 108.12%  (71.09%-152.67%) | 0.22  (-0.01-0.46) | 79.74%  (51.06%-112.59%) | -0.19  (-0.49-0.11) | 82.56%  (51.05%-120.65%) | -0.28  (-0.54--0.02) |
| Samoa | 136.63%  (51.35%-274.32%) | 0.95  (0.85-1.06) | 98.89%  (31.33%-210.62%) | 0.44  (0.40-0.48) | 97.11%  (23.89%-216.65%) | 0.43  (0.39-0.48) |
| San Marino | 133.30%  (63.69%-230.64%) | 1.33  (1.20-1.46) | 89.62%  (20.02%-192.40%) | 0.15  (-0.03-0.32) | 80.03%  (10.37%-185.20%) | 0.47  (0.29-0.65) |
| Sao Tome and Principe | 282.65%  (146.76%-475.59%) | 2.22  (2.05-2.39) | 194.36%  (91.71%-333.76%) | 1.55  (1.38-1.71) | 215.51%  (101.97%-384.19%) | 1.38  (1.14-1.61) |
| Saudi Arabia | 1029.97%  (574.90%-1764.18%) | 3.54  (3.34-3.73) | 351.76%  (173.06%-592.72%) | 0.48  (0.17-0.79) | 396.29%  (194.56%-676.48%) | 0.52  (0.25-0.80) |
| Senegal | 242.59%  (131.17%-399.98%) | 1.37  (1.20-1.54) | 204.39%  (108.07%-337.78%) | 1.01  (0.86-1.15) | 192.20%  (95.10%-325.91%) | 0.83  (0.66-1.01) |
| Serbia | 92.05%  (45.44%-154.90%) | 1.43  (1.25-1.61) | 48.94%  (14.81%-92.31%) | 0.24  (0.09-0.39) | 22.46%  (-6.89%-59.24%) | -0.23  (-0.43--0.03) |
| Seychelles | 339.94%  (248.04%-450.10%) | 2.46  (2.12-2.81) | 216.15%  (154.31%-288.59%) | 1.46  (1.16-1.78) | 214.80%  (149.77%-295.64%) | 1.26  (0.98-1.54) |
| Sierra Leone | 239.15%  (122.16%-389.38%) | 1.99  (1.88-2.10) | 190.40%  (96.70%-312.84%) | 1.58  (1.49-1.69) | 206.83%  (96.97%-349.44%) | 1.54  (1.44-1.64) |
| Singapore | 295.51%  (206.73%-400.89%) | 1.14  (0.83-1.43) | 151.20%  (128.70%-173.90%) | -0.86  (-1.04--0.67) | 116.65%  (96.68%-138.56%) | -1.07  (-1.25--0.90) |
| Slovakia | 97.70%  (46.90%-161.24%) | 0.96  (0.78-1.15) | 42.23%  (8.05%-85.14%) | -0.29  (-0.48--0.11) | 20.59%  (-9.44%-60.23%) | -0.81  (-0.96--0.65) |
| Slovenia | 41.73%  (-4.14%-105.71%) | -0.35  (-0.58--0.13) | 21.41%  (-15.47%-71.98%) | -1.57  (-1.84--1.29) | -4.59%  (-34.82%-39.19%) | -1.96  (-2.22--1.71) |
| Solomon Islands | 1491.62%  (928.33%-2622.87%) | 6.68  (6.04-7.33) | 1168.69%  (721.36%-2037.01%) | 5.97  (5.33-6.61) | 1269.16%  (783.90%-2207.72%) | 6.27  (5.58-6.96) |
| Somalia | 172.51%  (69.82%-321.19%) | 0.58  (0.50-0.66) | 169.67%  (75.49%-314.61%) | 0.54  (0.47-0.61) | 159.83%  (64.34%-311.89%) | 0.35  (0.30-0.41) |
| South Africa | 147.86%  (114.12%-185.26%) | 0.97  (0.78-1.17) | 124.01%  (98.11%-157.57%) | 0.57  (0.29-0.85) | 94.76%  (68.33%-125.42%) | 0.23  (-0.03-0.49) |
| South Sudan | 94.35%  (29.64%-181.22%) | 0.45  (0.26-0.63) | 77.82%  (18.30%-156.72%) | 0.19  (0.03-0.36) | 86.43%  (18.88%-183.08%) | 0.20  (0.01-0.40) |
| Spain | 74.45%  (36.09%-124.22%) | -0.04  (-0.17-0.09) | 23.77%  (12.62%-33.86%) | -1.70  (-1.82--1.57) | 3.14%  (-5.14%-11.23%) | -1.90  (-2.04--1.76) |
| Sri Lanka | 330.93%  (204.39%-498.94%) | 3.12  (2.95-3.28) | 205.65%  (117.70%-313.00%) | 1.69  (1.53-1.85) | 157.23%  (79.06%-253.67%) | 1.35  (1.21-1.49) |
| Sudan | 283.71%  (138.81%-496.87%) | 1.78  (1.59-1.96) | 169.71%  (75.03%-314.91%) | 0.79  (0.67-0.93) | 175.45%  (74.69%-328.12%) | 0.73  (0.59-0.87) |
| Suriname | 223.00%  (147.90%-313.04%) | 1.18  (0.92-1.43) | 169.90%  (110.55%-236.25%) | 0.58  (0.34-0.82) | 159.80%  (98.64%-236.28%) | 0.45  (0.21-0.68) |
| Sweden | 42.60%  (13.56%-77.89%) | 0.19  (-0.03-0.42) | 17.05%  (8.57%-26.50%) | -0.74  (-0.83--0.66) | 1.85%  (-5.55%-10.01%) | -1.01  (-1.13--0.90) |
| Switzerland | 19.49%  (-8.74%-55.48%) | -0.94  (-1.09--0.77) | -13.10%  (-21.06%--5.59%) | -2.17  (-2.35--1.98) | -21.88%  (-28.82%--14.26%) | -2.40  (-2.53--2.26) |
| Syrian Arab Republic | 343.01%  (168.23%-614.99%) | 2.40  (2.10-2.71) | 201.37%  (83.26%-387.06%) | 0.82  (0.49-1.16) | 180.82%  (70.74%-348.82%) | 0.66  (0.30-1.03) |
| Taiwan  (Province of China) | 370.92%  (254.84%-530.66%) | 3.15  (2.70-3.61) | 214.34%  (143.82%-309.42%) | 1.22  (1.02-1.41) | 172.70%  (108.48%-260.86%) | 1.13  (0.91-1.35) |
| Tajikistan | 121.70%  (68.40%-189.75%) | 0.19  (-0.07-0.46) | 88.79%  (44.03%-144.64%) | 0.05  (-0.17-0.27) | 94.53%  (47.14%-154.68%) | -0.46  (-0.65--0.26) |
| Thailand | 303.36%  (183.46%-465.16%) | 1.92  (1.36-2.48) | 173.16%  (95.97%-272.70%) | 0.13  (-0.32-0.58) | 139.31%  (67.36%-236.16%) | 0.10  (-0.41-0.62) |
| Timor-Leste | 254.93%  (124.17%-417.68%) | 1.79  (1.43-2.15) | 198.61%  (100.54%-330.61%) | 0.96  (0.65-1.29) | 159.02%  (56.32%-282.15%) | 0.80  (0.46-1.15) |
| Togo | 292.72%  (171.20%-460.01%) | 1.07  (0.92-1.22) | 253.46%  (151.93%-399.14%) | 0.81  (0.69-0.92) | 239.16%  (132.21%-390.58%) | 0.52  (0.37-0.66) |
| Tokelau | 61.90%  (7.48%-164.28%) | 1.48  (1.39-1.57) | 19.87%  (-17.74%-87.24%) | 0.57  (0.46-0.67) | 20.21%  (-20.17%-96.06%) | 0.35  (0.28-0.43) |
| Tonga | 77.05%  (24.49%-158.81%) | 0.66  (0.53-0.80) | 61.99%  (17.94%-128.11%) | 0.32  (0.21-0.42) | 45.15%  (0.86%-113.01%) | 0.05  (-0.08-0.17) |
| Trinidad and Tobago | 125.49%  (62.82%-203.60%) | 0.27  (0.10-0.44) | 88.14%  (40.43%-147.68%) | -0.64  (-0.77--0.51) | 72.37%  (24.66%-130.65%) | -0.67  (-0.81--0.52) |
| Tunisia | 356.55%  (193.46%-573.50%) | 2.09  (1.96-2.21) | 190.88%  (93.18%-318.21%) | 0.35  (0.23-0.47) | 178.84%  (81.08%-302.95%) | 0.41  (0.29-0.53) |
| Turkey | 320.55%  (195.61%-484.82%) | 2.56  (2.26-2.87) | 140.43%  (71.93%-229.07%) | 0.33  (0.06-0.59) | 113.23%  (51.12%-194.78%) | -0.03  (-0.26-0.19) |
| Turkmenistan | 162.87%  (103.50%-243.41%) | 1.10  (0.55-1.64) | 94.53%  (50.17%-150.07%) | -0.16  (-0.64-0.32) | 99.44%  (53.68%-158.66%) | 0.03  (-0.49-0.55) |
| Tuvalu | 90.58%  (29.59%-182.08%) | 0.82  (0.71-0.92) | 63.55%  (13.18%-138.84%) | 0.27  (0.20-0.35) | 48.19%  (1.51%-117.23%) | 0.10  (0.04-0.16) |
| Uganda | 281.54%  (162.06%-453.00%) | 1.25  (1.05-1.43) | 214.68%  (119.77%-346.86%) | 0.66  (0.48-0.84) | 239.88%  (129.29%-403.52%) | 0.77  (0.52-1.02) |
| Ukraine | -2.60%  (-24.12%-23.87%) | -0.82  (-1.09--0.55) | -16.09%  (-33.53%-3.53%) | -1.64  (-1.96--1.32) | -21.40%  (-37.75%--2.33%) | -1.80  (-2.16--1.45) |
| United Arab Emirates | 1302.29%  (795.29%-2021.15%) | 0.23  (-0.50-0.97) | 850.91%  (512.54%-1335.31%) | -0.80  (-1.63-0.04) | 933.61%  (565.88%-1445.17%) | -0.53  (-1.14-0.10) |
| United Kingdom | 26.02%  (0.01%-57.38%) | -0.39  (-0.47--0.30) | -12.48%  (-16.70%--8.23%) | -2.04  (-2.16--1.92) | -20.32%  (-23.61%--16.46%) | -2.14  (-2.25--2.01) |
| United Republic of Tanzania | 244.91%  (154.37%-365.85%) | 1.37  (1.27-1.46) | 201.56%  (127.47%-301.84%) | 0.92  (0.85-1.01) | 203.10%  (120.99%-319.17%) | 0.96  (0.86-1.07) |
| United States of America | 33.28%  (10.16%-61.52%) | -1.09  (-1.20--0.99) | 14.12%  (9.23%-18.83%) | -1.71  (-1.80--1.61) | 4.81%  (-0.15%-9.84%) | -1.85  (-1.96--1.74) |
| United States Virgin Islands | 110.89%  (58.61%-185.82%) | 0.71  (0.60-0.82) | 89.96%  (47.13%-145.49%) | -0.04  (-0.11-0.04) | 53.26%  (15.18%-107.79%) | -0.27  (-0.32--0.23) |
| Uruguay | 38.16%  (7.45%-75.85%) | 0.02  (-0.09-0.14) | 16.01%  (6.09%-25.90%) | -0.90  (-0.97--0.82) | -1.10%  (-9.18%-7.33%) | -1.11  (-1.17--1.06) |
| Uzbekistan | 231.82%  (167.05%-307.14%) | 1.15  (0.95-1.34) | 146.42%  (100.69%-199.10%) | 0.55  (0.33-0.79) | 164.97%  (114.20%-223.33%) | 0.25  (0.02-0.49) |
| Vanuatu | 373.62%  (185.71%-777.23%) | 1.97  (1.76-2.18) | 338.69%  (172.53%-697.62%) | 1.72  (1.55-1.91) | 326.75%  (155.91%-689.64%) | 1.69  (1.49-1.90) |
| Venezuela  (Bolivarian Republic of) | 403.10%  (270.03%-570.55%) | 2.10  (1.97-2.25) | 248.90%  (162.65%-357.52%) | 0.64  (0.51-0.78) | 220.49%  (136.93%-329.99%) | 0.62  (0.47-0.76) |
| Viet Nam | 341.60%  (206.41%-518.20%) | 2.29  (2.13-2.44) | 177.83%  (95.10%-276.19%) | 0.72  (0.62-0.83) | 178.00%  (90.37%-288.36%) | 0.64  (0.51-0.77) |
| Yemen | 419.04%  (225.61%-821.81%) | 2.29  (2.15-2.42) | 308.42%  (155.86%-611.44%) | 1.38  (1.27-1.49) | 321.70%  (162.73%-650.00%) | 1.46  (1.36-1.57) |
| Zambia | 215.00%  (115.80%-342.62%) | 0.69  (0.58-0.79) | 159.36%  (82.15%-259.12%) | 0.18  (0.09-0.27) | 156.86%  (73.46%-269.16%) | -0.09  (-0.20-0.03) |
| Zimbabwe | 197.02%  (99.42%-331.54%) | 2.37  (1.94-2.79) | 194.94%  (98.36%-323.55%) | 2.91  (2.35-3.47) | 203.30%  (98.99%-341.94%) | 3.22  (2.57-3.88) |

Abbreviations: ASIR, age-standardized incident rates. ASMR, age-standardized mortality rates. CI, confidential interval. DALY, disability adjusted life-year. EAPC, estimated annual percentage change. UI, uncertain interval.
