# Supplementary material for: The Global, Regional, and National Burden and Trends of Breast Cancer From 1990 to 2019: Results From the Global Burden of Disease Study 2019
Source: Front Oncol. 2021 May 21;11:689562. doi: 10.3389/fonc.2021.689562 (PMC8176863; doi:10.3389/fonc.2021.689562)
Supplement: Supplementary file 7 [file Table_3.docx]

**Supplementary Table 3 Four prevalence sequelae by breast cancer in GBD study**

Total prevalence for each cancer is split into the following four health state: 1. diagnosis and primary therapy; 2. controlled phase; 3. metastatic phase; and 4. terminal phase. The diagnosis and primary therapy phase are defined as the time from the onset of symptoms to the end of treatment. The controlled phase is defined as the time between finishing primary treatment and the earliest of either: cure (defined as recurrence- and progression-free survival after 10 years); death from another cause; or progression to the metastatic phase. The metastatic phase is defined as the time period of intensive treatment for metastatic disease, as determined for each cancer by SEER (Surveillance, Epidemiology, and End Results Program) averages. The terminal phase is defined as the one-month period prior to death. What’s more, additional disability beyond these four sequelae is estimated for breast cancer (disability due to mastectomy) for the long-term disability associated with treatment-related Procedures.

**Duration of four prevalence sequelae by cancer**

|  | Diagnosis/Treatment(months) | Remission | Disseminated/metastatic  (months) | Note | Terminal (months) |
| --- | --- | --- | --- | --- | --- |
| Breast cancer | 3 | Calculated based on remainder of time after attributing other sequelae | 17.7 | SEER Summary Stage 1997 (Distant site/node involved) 1995-2000 | 1 |

**GBD 2019 sequelae, health states, health state lay descriptions, and disability weights**

| health state |  | Health state lay description | Disability Weight |
| --- | --- | --- | --- |
| Diagnosis and primary therapy phase | Cancer, diagnosis and primary therapy | has pain, nausea, fatigue, weight loss and high anxiety | 0.288  (0.193-0.399) |
| Metastatic phase | Cancer, metastatic | has severe pain, extreme fatigue, weight loss and high anxiety. | 0.451  (0.307-0.6) |
| Mastectomy from breast cancer, beyond 10 years | Mastectomy | had one of her breasts removed and sometimes has pain or swelling in the arms. | 0.036  (0.02-0.057) |
| Terminal phase | Terminal phase, with medication (for cancers, end-stage kidney/liver disease) | has lost a lot of weight and regularly uses strong medication to avoid constant pain. The person has no appetite, feels nauseous, and needs to spend most of the day in bed | 0.54  (0.377-0.687) |
| Controlled phase, without mastectomy | Generic uncomplicated disease: worry and daily medication | has a chronic disease that requires medication every day and causes some worry but minimal interference with daily activities | 0.049  (0.031-0.072) |
| Controlled phase, with mastectomy | Mastectomy and generic medication | (combined DW) | 0.083  (0.052-0.124) |
